# Identification of Genes Encoding Antimicrobial Proteins in Langerhans Cells

**DOI:** 10.3389/fimmu.2021.695373

**Published:** 2021-08-26

**Authors:** Aislyn Oulee, Feiyang Ma, Rosane M. B. Teles, Bruno J. de Andrade Silva, Matteo Pellegrini, Eynav Klechevsky, Andrew N. Harman, Jake W. Rhodes, Robert L. Modlin

**Affiliations:** ^1^Division of Dermatology, Department of Medicine, University of California, Los Angeles, Los Angeles, CA, United States; ^2^Department of Microbiology, Immunology and Molecular Genetics, University of California, Los Angeles, Los Angeles, CA, United States; ^3^Department of Molecular, Cell and Developmental Biology, University of California, Los Angeles, Los Angeles, CA, United States; ^4^Department of Pathology and Immunology, Washington University School of Medicine, St. Louis, MO, United States; ^5^Centre for Virus Research, The Westmead Institute for Medical Research, Westmead, NSW, Australia; ^6^School of Medical Sciences, Faculty of Medicine and Health Sydney, The University of Sydney, Westmead, NSW, Australia

**Keywords:** Langerhans cells, dendritic cells, antimicrobial peptides, immunity, skin, transcriptome, bioinformatics

## Abstract

Langerhans cells (LCs) reside in the epidermis where they are poised to mount an antimicrobial response against microbial pathogens invading from the outside environment. To elucidate potential pathways by which LCs contribute to host defense, we mined published LC transcriptomes deposited in GEO and the scientific literature for genes that participate in antimicrobial responses. Overall, we identified 31 genes in LCs that encode proteins that contribute to antimicrobial activity, ten of which were cross-validated in at least two separate experiments. Seven of these ten antimicrobial genes encode chemokines, *CCL1, CCL17, CCL19, CCL2, CCL22, CXCL14* and *CXCL2*, which mediate both antimicrobial and inflammatory responses. Of these, *CCL22* was detected in seven of nine transcriptomes and by PCR in cultured LCs. Overall, the antimicrobial genes identified in LCs encode proteins with broad antibacterial activity, including against *Staphylococcus aureus*, which is the leading cause of skin infections. Thus, this study illustrates that LCs, consistent with their anatomical location, are programmed to mount an antimicrobial response against invading pathogens in skin.

## Introduction

Dendritic cells (DCs) are the key antigen presenting cells (APCs) that control both immunity and tolerance (1). DCs are localized in most tissues and surface barriers, where they function as sentinels for pathogen recognition. Stimulation of innate signaling receptors induce DCs to migrate from the periphery to secondary lymphoid organs, where they present antigens that drive adaptive immunity. DCs are divided into distinct subsets characterized by their unique expression of surface receptors and transcription factors, pathogen sensors and cytokines secretion profiles that contribute to their specialized capacities in activating different modules of immunity (2–6).

Human skin harbors multiple types of dendritic-appearing cells including Langerhans cells (LCs) that exclusively reside in the epidermis as well as conventional DCs (cDCs) in the underlying dermis. In addition to their localization to the epidermis, LCs are distinguished by their high expression of CD1a, the C-type lectin langerin (CD207) which induces the formation of a LC-specific organelle, the Birbeck granule, and lower expression of CD11c than dermal DCs (7). Through their dendrites, they form an extensive cellular network patrol the interface between the skin and the outside environment for pathogens (8–10), bind microbial ligands *via* toll-like receptors (TLRs) and CD207 and taking up pathogens *via* endocytosis (11–13).

LCs, although derived in mice from similar precursors as macrophages, have antigen presentation capacities similar to DCs (14, 15). Upon antigen capture, LCs undergo phenotypic changes during maturation and migrate to regional lymph nodes where they activate adaptive responses (12, 16–18). During infection, LC emigration from the epidermis is significantly enhanced by inflammatory cytokines such as IL-1 and TNF (19, 20). Migrating LCs express the DC-specific transcription factor ZBTB46 (21, 22), IL-15 (23) and IRF4, which is important for their ability to prime and cross-present antigens to CD8^+^ T cells at that site (24, 25). Additionally, LCs enhances cellular immunity by inducing Th1 and Th2 differentiation of CD4^+^ T cells (3, 4), they are the main skin DC subset responsible for directing IL-17 and IL-22-mediated responses (12, 26, 27), indicative of skin inflammatory and antimicrobial diseases. A subset of migratory LCs express CD5 with an even greater capacity to amplify these T cell responses (6). Moreover, a unique aspect of human LC is their ability to present antigen *via* CD1a both autoreactive (28, 29) and *Mycobacterium leprae*- and *M. tuberculosis*-reactive CD1a-restricted T cell responses have been reported (30).

Although it has been previously shown that LCs contribute to cutaneous host defense against pathogens including viruses (13, 31, 32), bacteria and fungi (33), only a few genes have been identified that directly mediate the antimicrobial response. In order to more broadly define the mechanisms by which LCs potentially contribute to an antimicrobial response, we mined public LC transcriptomes and surveyed the literature to identify “antimicrobial genes”, defined as genes encoding proteins with direct antimicrobial activity.

## Materials and Methods

### Gene Expression Omnibus (GEO) Analysis

We surveyed Gene Expression Omnibus (GEO) (34) for transcriptomes of human skin-derived LCs and Langerhans-like dendritic cells (LCDCs), which are derived from CD34^+^ stem cells, using the key terms “(Langerhans AND skin) AND Homo sapiens[Organism]”. Our search was for the period before August 2020 and include those studies in which the LCs were activated with pro-inflammatory stimuli and/or as compared to other myeloid populations. This search yielded 24 series, nine of which met the criteria that n ≥ 3 samples for the LC and comparison group and did not contain only Langerhans cell histiocytosis samples. We then used GEO2R, an R-based web application, to obtain a list of genes that were differentially expressed in LCs. After obtaining the list of genes, we then filtered the comparisons by logFC>1 and adj. p-value<0.05. Of the nine series which the described criteria, one (GSE32648) did not yield any recognizable gene names on GEO2R and therefore we contacted the authors who provided us with their new RNA-seq data instead of the microarray data currently deposited in GEO2R and for a second dataset (GSE120386), GEO2R was not available. We used DESeq2 to run differential expression analysis of both of the bulk RNA-seq data with the default parameters. Genes with an adj. p value <0.05 were considered significantly differentially expressed.

### LC Antimicrobial Genes

We curated our direct antimicrobial list based on the 105 antimicrobial peptides listed in the Antimicrobial Peptide Database (APD) (35). The criteria for data registration into APD are the following: the peptides must be from natural sources, their antimicrobial activities must have been demonstrated (MIC <100 ug/ml), and their amino acid sequences elucidated. We also supplemented this list with literature findings of eight genes encoding peptides with direct antimicrobial activity not yet registered into the database including *CCL2* (36)*, CCL14*, *CCL15* (37), *CXCL7* (38), *CXCL17* (39), *MPEG1* (40), *S1008A* (41), and *S1009A* (42) yielding a total of 113 genes. To identify which genes encoded peptides with direct antimicrobial activity, we overlapped the results with our curated direct antimicrobial list using Venny 2.1 (43).

We also reviewed the literature for direct antimicrobial genes using the key terms “(Langerhans [Title/Abstract]) AND (antimicrobial [Title/Abstract])” which yielded 40 results of which five studies contained evidence for eight genes encoding peptides with direct antimicrobial activity in LCs. Our search “(Langerhans [Title/Abstract]) AND (antibacterial [Title/Abstract])” yielded 21 results, none of which included genes encoding peptides with direct antimicrobial in LCs.

### Ingenuity Pathway Analysis (IPA) Upstream Regulator Prediction

IPA Upstream Regulator Analysis was used to identify upstream regulators and predict whether they are activated or inhibited, given the observed gene expression changes in our experimental dataset. The analysis examines the known targets of each upstream regulator in a dataset, compares the targets’ actual direction of change to expectations derived from the literature, then generates a prediction for each upstream regulator. Briefly, IPA uses an ‘enrichment’ score [Fisher’s exact test (FET) P-value] that measures the overlap of observed and predicted regulated gene sets.

## Results

### Identification and Characteristics of Langerhans Cells Transcriptomes

To identify potential mechanisms by which LCs mount an antimicrobial response, we queried GEO and identified seven microarray series that permitted the mining of the LC transcriptome data using GEO2R. In addition, there was one bulk RNA-seq series for which GEO2R was not available (GSE120386) and another bulk-RNA seq data series not yet deposited in GEO2R and therefore we used DESeq2 on RStudio to compute the differential gene expression for both data series (**Supplementary Table S1**).

In three of nine series, LCs were directly isolated from skin specimens by enzymatic digestion, and the transcriptomes measured immediately. In one study, CD11c^+^ DDCs were directly isolated from skin and monocyte derived DCs and CD1c^+^ DCs from blood (44). In another study, plasmacytoid DCs (pDCs) and myeloid DCs (mDCs) were isolated from peripheral blood (45), and in the third study, pDCs were isolated from spleen and dermal macrophages from skin (46). Five of the nine transcriptomes were derived from LCs isolated by migration, in order to represent those LCs that are in the process of migration to lymph nodes, albeit this leads to an altered phenotype. In one study each, CD14^+^ DCs and CD14^+^ macrophages (47), or CD141^+^ dermal DCs, CD14^+^ dermal DCs, and CD141^-^CD14^-^ dermal DCs (21), or dermal langerin^-^ type 2 conventional dendritic cell (cDC2), dermal langerin^+^ cDC2, dermal CD14^+^CD1c^-^ monocyte-derived macrophages, and dermal CD14^+^CD1c^+^ monocyte-derived dendritic cells (48) were isolated from skin by enzymatic digestion. In two of the migratory LC studies, the LC transcriptomes were measured at time zero and various timepoints following stimulation by TNF at (24, 49). In the same transcriptome, CD11c^+^ dermal DCs were also isolated by migration (49). In the last series, Langerhans-like dendritic cells (LCDCs) were generated *in vitro* and infected with the live mosquito-derived third-stage larvae (L3) of the parasitic nematode *Brugia malayi* (50).

### Identification of Antimicrobial Genes in LCs

We mined the LC transcriptomes by comparing either LCs to another myeloid cell type or a specific time point following stimulation. We filtered the comparisons by logFC>1 and adjusted p-value <0.05, then overlapped the results with the direct antimicrobial gene list consisting of 113 genes using Venny 2.1 (43). Using this approach, we identified 23 genes encoding proteins with direct antimicrobial activity in the LC transcriptomes (**Table 1**). Of these 23 genes, 11 were uniquely identified in LCs isolated by migration (then either unstimulated or cytokine activated), nine were uniquely identified in LCs derived from digested skin samples and three were presented in LCs isolated by migration as well as from digested skin samples. Although there were more genes identified in LCs obtained by migration from skin samples as compared to digested skin samples, as LCs isolated by enzymatic digestion are immature compared to those isolated by migration which are in a mature state, and that the migrated LCs were sometimes activated with cytokines whereas the digested LCs were not (55).

**Table 1:** Identification of genes encoding peptides with antimicrobial activity in LCs in transcriptome studies.

Genes	Multiple transcriptomes			Identified in the literature in non-transcriptome studies	References	Total instances identified
	Number of transcriptome studies	Number of transcriptome comparisons	Comparisons			
*CCL22*	6	7	LCs vs pDCs (T2, T8), LCs vs CD11c+ dermal DCs (T4), LCs vs dermal MФ (T5), LCs vs CD14+ dermal DCs (T6), LCs vs CD14+ CD1c-monocyte-derived MФ (T9) and LCs vs monocyte-derived CD14+CD1c+DCs (T9)	1	[Ross et al. (51)]	8
*CXCL14*	3	6	LCs vs CD1c+ mDCs (T1 and T2), LCs vs pDCS (T2), LCs vs Dermal langerin- cDC2 (T9), LCs vs CD14+ CD1c-monocytederived MФ (T9) and LCs vs monocyte- derived CD14+CD1c+DCs (T9)	0	N/A	6
*ADM*	3	4	LCs vs CD1c+ mDCs (T2), LCs vs pDCS (T2), LCs 2h vs 0h (T4, T7)	0	N/A	4
*CCL20*	2	4	LCs vs moDCs (T1), LCs vs blood CD1c+ mDCs (T1), LCs at 2h vs 0h (T7), LCs 24h vs 0h (T7)	0	N/A	4
*B2M*	2	3	LCs vs CD1c+ mDCs (T2), LCs vs CD141+ dermal DCs (T6), LCs vs CD14+ dermal DCs (T6)	0	N/A	3
*CCL17*	1	2	LCs 24h vs 0h (T4), LCs 8h vs 0h (T4)	1	[Alferink et al. (52)]	3
*CCL19*	2	3	LCs 8h vs 0h (T4), LCs 24h vs 0h (T4, T7)	0	N/A	3
*CXCL2*	2	2	LCs vs blood CD1c^+^ mDCs (T1), LCs 2h vs 0h (T7)	1	[Heufler et al. (53)]	3
*CCL1*	1	1	LCs 24h vs 0h (T4)	1	[Schaerli et al. (54)]	2
*CCL2*	2	2	LCs 24h vs 0h (T4 and T7)	0	N/A	2
*CCL27*	1	2	LCs vs moDCs (T1), LCs vs blood CD1c+ mDCs (T1), LCs 24h vs 0h, LCs 8h vs 0h (T4)	0	N/A	2
*DEFB1*	1	2	LCs vs moDCs (T1), LCs vs blood CD1c+ mDCs (T1)	0	N/A	2
*FURIN*	1	2	LCs vs pDCs (T8), LCs vs MФ (T8)	0	N/A	2
*GAPDH*	2	2	LCs vs pDCs (T2 and T8)	0	N/A	2
*HMGN2*	1	2	LCs vs CD141+ Dermal DCs (T6), LCs vs CD141-CD14- DCs (T6)	0	N/A	2
*LEAP2*	1	2	LCs vs Dermal CD14+ CD1c+ DCs (T9), LCs vs Dermal Langerin+ cDC2 (T9)	0	N/A	2
*S1007A*	1	2	LCs vs moDCs (T1), LCs vs blood CD1c+ mDCs (T1)	0	N/A	2
*SNCA*	1	2	LCs vs moDCs (T1), LCs vs blood CD1c+ mDCs (T1)	0	N/A	2

By mining publicly available data in GEO DataSets, we were able to identify 23 genes encoding antimicrobial peptides that are more strongly expressed in LCs vs. other DC subtypes and/or in LCs activated with cytokines vs. LCs with no activation. N/A, Not applicable.

### Antimicrobial Genes Upregulated in Activated LCs and in LCs Compared to Other Cell Types

**Figure 1:**
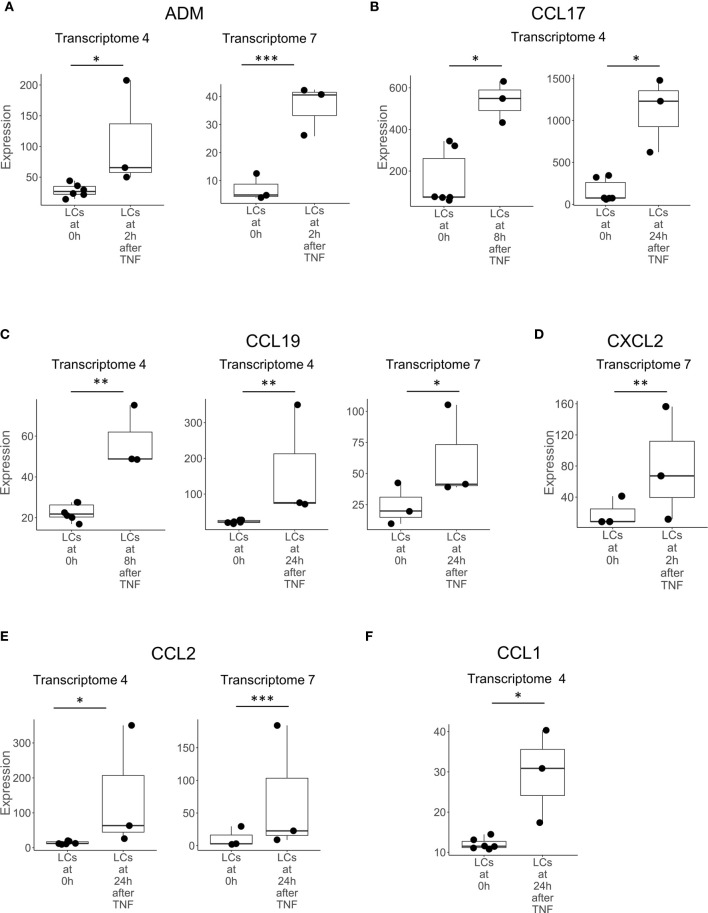
Genes upregulated in LCs after activation with TNF. Boxplots showing the expression of **(A)** *ADM*, **(B)** *CCL17*, **(C)** *CCL19*, **(D)** *CXCL2*, **(E)** *CCL2*, and **(F)** *CCL1*, encoding antimicrobial peptides in LCs prior to (0h) and at various time points following activation with TNF in transcriptomes 4 and 7. Genes shown above were identified in at least two instances, either in multiple transcriptomes experiments or in one transcriptome experiment but also identified in LCs in non-transcriptome experiments in the literature. *p < 0.05, **p < 0.01, ***p < 0.001.

We examined the transcriptomes of LCs activated *in vitro* by cytokines or microbes. We identified eight genes that encode proteins with direct antimicrobial activity by mining the two transcriptomes of TNF-activated migratory LCs (Transcriptomes 4 and 7), this was the greatest number in any of the comparisons performed (**Supplementary Table S2**). There were six genes encoding chemokines that were upregulated in migratory LCs after stimulation with TNF: *CCL1, CCL2, CCL17, CCL19, CCL20*, and *CXCL2.* In addition, we detected two other genes, *ADM* and *IL26* in LCs stimulated with TNF (**Figure 1**). Of the eight total genes, *CCL2*, *CCL19* and *ADM* were detected in both transcriptomes of TNF treated LCs. We did not identify any genes encoding peptides with direct antimicrobial genes upregulated in LCs stimulated with live mosquito-derived third-stage larvae (L3) of *B. malayi*, which is consistent with the previous finding that the live mosquito-derived third-stage larvae (L3) fails to activate LCs compared to known activators (50). By analyzing the comparisons of LCs to other cell types, we identified 16 antimicrobial genes, of which only *ADM* was identified in the transcriptomes of TNF treated LCs.

We found nine studies in which the LC transcriptome was compared to other DC subtypes, including dermal DCs, peripheral blood DCs and cytokine-derived DCs, as well as to macrophage subpopulations. The nomenclature used to define DC subpopulations has evolved with changing technologies, such that different studies use different markers to define subpopulations. Dermal DCs have been identified based on the expression of various cell surface markers including XCR1^+^, CD141^+^, CD1c^+^, CD1a^+^ and CD14^+^ (3, 21, 47, 56–58), which may vary according to the method of isolation, digestion vs. migration (55). The analysis of DC subpopulations in human blood by single cell RNA sequencing has led to a revised gene-based classification (59). In reporting the comparison of transcriptomes in LCs to other cell types, we have maintained the nomenclature in the original citation.

**Figure 2:**
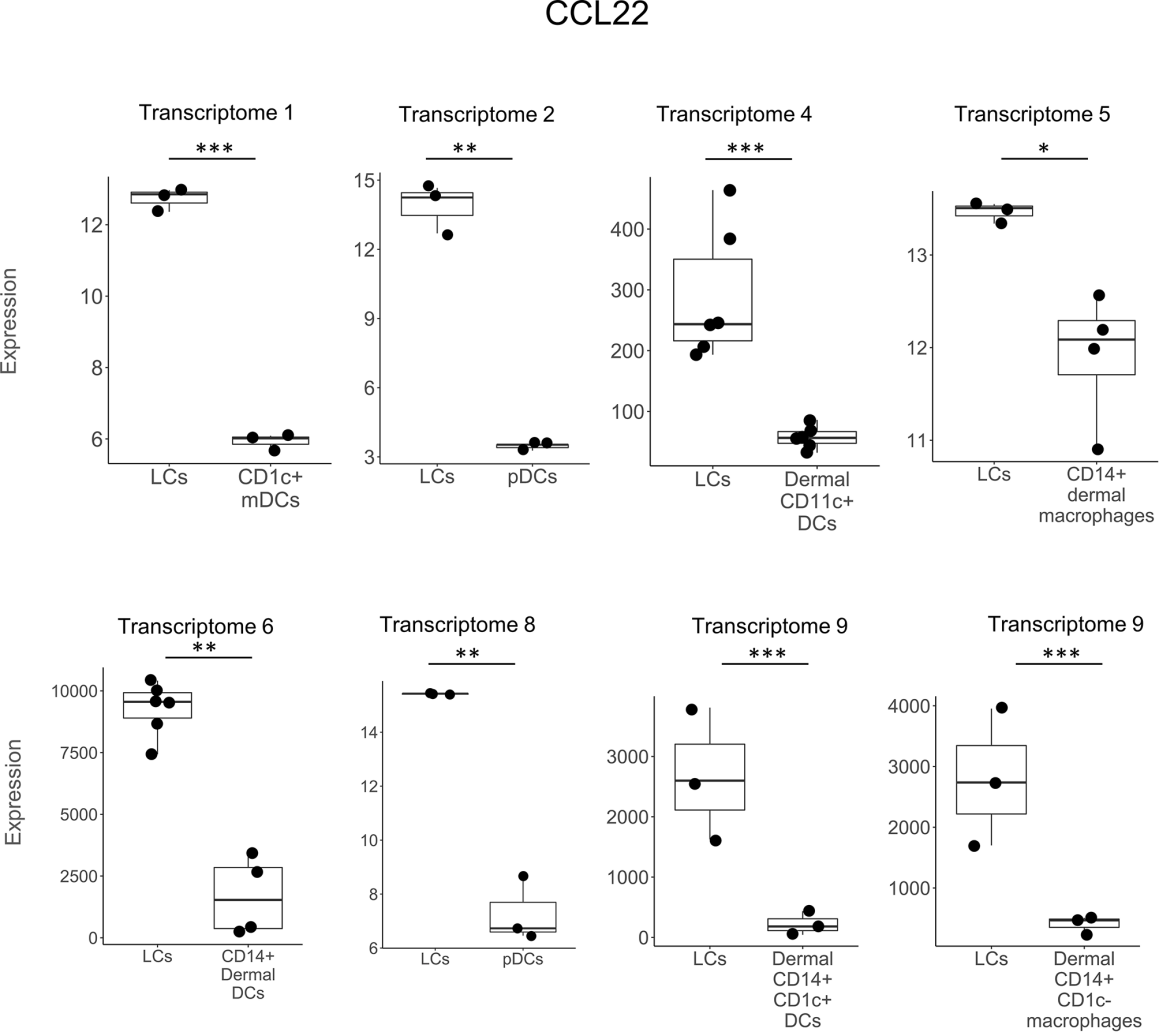
*CCL22* expression in LCs vs other DC subtypes. *CCL22* was preferentially expressed in LCs vs other DC types in 7 out of the 9 transcriptomes in a total of 8 instances. *CCL22* was the most frequently detected gene in transcriptomes and was also previously reported in LCs in non-transcriptome experiments in the literature. *p < 0.05, **p < 0.01, ***p < 0.001.

**Figure 3:**
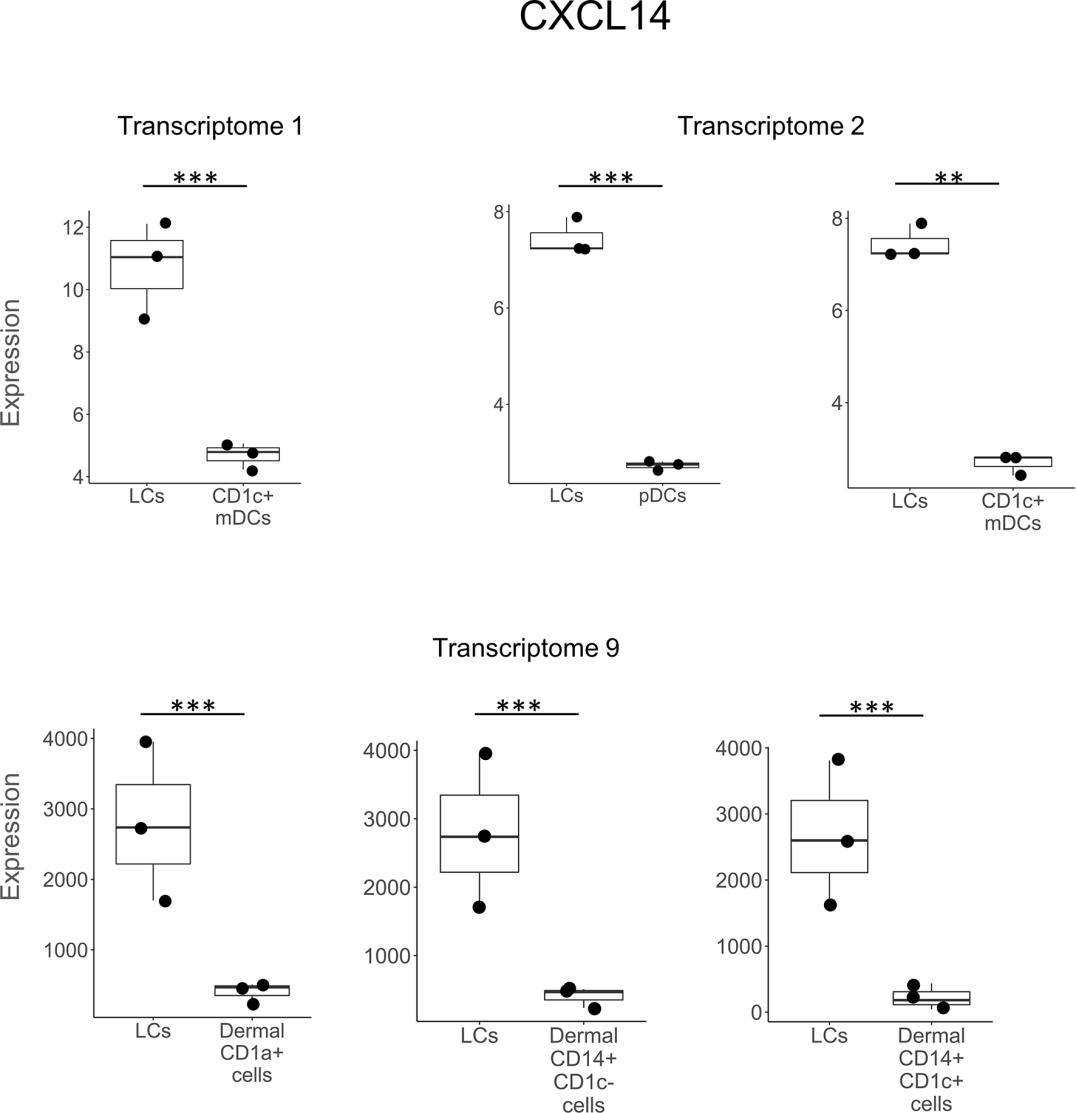
*CXCL14* expression in LCs vs other DC subtypes. *CXCL14* was preferentially expressed in LCs vs other DC types in 3 out of the 9 transcriptomes in a total of 6 different instances. **p < 0.01, ***p < 0.001.

In comparing LCs to other DC and myeloid cell types, *CCL22* was the most frequently detected gene, expressed in seven of the nine studies and in eight separate comparisons (**Figure 2**). *CXCL14* was detected as upregulated in six instances in three LCs transcriptomes (**Figure 3**). *B2M* was identified in the transcriptomes of LCs compared to other cell types in three different instances (**Supplementary Figure S1**). *GAPDH* was more highly expressed in LCs in two different transcriptomes (**Supplementary Figure S2**). *CCL27*, *DEFB1, FURIN, LEAP2, SNCA*, and *S100A7* were each identified as preferentially expressed in LCs in two instances but always in a single LC transcriptome as compared to other cell types (**Supplementary Table S3**). *HMGN2* was preferentially expressed in LCs compared to CD141^+^ and CD141^-^CD14^-^ dermal DCs in one transcriptome (**Supplementary Figure S3**). *SAA2, FAM3A*, and *RARRES2* were identified as upregulated in LCs in one instance each. Heat maps showing the expression of each gene in the different transcriptome comparisons are shown in **Supplementary Figures 4–6**.

### Antimicrobial Genes in LCs Identified in the Literature

We found corroborating evidence in the literature that four of the 23 direct antimicrobial genes were expressed in LCs. These included the *CCL17*-encoded peptide in cytokine activated LCs (52)*, CXCL2* mRNA in freshly isolated LCs (53), *CCL22* mRNA during maturation of LCs (51), and the *CCL1*-encoded peptide in epidermal LCs *in situ* (54). We found reports indicating expression of eight genes encoding directly antimicrobial peptides and/or the antimicrobial proteins themselves in activated LCs that were not detected in any of transcriptomes. These include *CXCL9, CXCL10*, *CXCL11* (60), *POMC* (61) and *NPY* (62) mRNAs, as well as *CAMP*, *DEFB4* (33) and *DEFB103* (63, 64) encoded antimicrobial peptides (**Supplementary Table S4**). Thus, a total of 31 antimicrobial genes/proteins were identified in LCs from analysis of LCs transcriptomes and published studies.

### Cross-Validation of Antimicrobial Genes

Overall, we found that ten of the 23 antimicrobial genes identified in the LC transcriptomes were cross-validated in at least two separate studies in the nine LC transcriptomes and/or four additional published studies. Six of the ten antimicrobial genes were cross validated by detection in two separate LC transcriptomes each, in each instance comparing LCs to the same other DC or myeloid cell type. *CXCL14* was upregulated in LCs vs. blood CD1c+ DCs (Transcriptomes 1 and 2), *CCL22* and GAPDH in LCs vs pDCs (Transcriptomes 2 and T8), and *B2M* in LCs compared to different DC populations in Transcriptomes 2 and 6. *CCL2* and *CCL19* were each upregulated in LCs treated with TNF for 24 hours vs 0 hours (Transcriptomes 4 and 7). *ADM* was upregulated in LCs treated with TNF for 2 hours vs 0 hours (Transcriptomes 4 and 7) and was also more strongly expressed in LCs vs blood CD1c+ DCs. *CCL22* and *CXCL2* expression was greater in LCs compared to other cell types in seven and two different transcriptomes, respectively, and validated by reverse transcriptase-polymerase chain reaction in additional studies (51, 53).

We also examined which antimicrobial genes were differentially expressed in LCs vs keratinocytes (KCs). We surveyed GEO DataSets for datasets containing both LCs and KCs using the key terms “Langerhans AND keratinocytes” and found two datasets (GSE168167 and GSE72104), both data sets containing LCs (n=3) and KCs (n=2) although our original criteria required n≥3 for each cell type. We found the expression of *CCL22* was greater in LCs than KCs for both datasets, showing a 6.4- and 3.9-fold change. In one dataset (GSE72104), *CCL17* expression was 4.3-fold greater in LCs than KCs and was identified in transcriptome 4 as being upregulated in LCs by TNF at 8 and 24 hours and validated at the protein level in a reporter mouse (*CCL17*) (52). *CCL1* was identified in a single LC transcriptome upregulated by TNF after 24 hours and the protein validated by immunohistochemistry (*CCL1*) (54).

Using Ingenuity Pathways Analysis, we investigated the canonical pathways in LCs compared to other cell types, focusing on the three “Noah’s ark like” instances in which LCs were compared to the identical cell type in two transcriptomic studies. Thus, there were two studies each comparing LCs to pDCs, blood CD1c+ DCs and CD14^+^ dermal DCs. From the top 100 canonical pathways in each comparison, we identified one pathway present in all six comparisons and 23 in 5/6 comparisons (**Supplementary Table S7**), noting that there were fewer genes and hence pathways identified in LCs vs. CD14+ dermal DCs from Transcriptome 5. Overall, 21/23 pathways were identified as “signaling” pathways, including RANK, CD40, CXCR4, IL6 and IL8 signaling, consistent with the known functional properties of LCs.

### Upstream Regulator Analysis of Genes Encoding Antimicrobial Proteins in LCs

**Figure 4:**
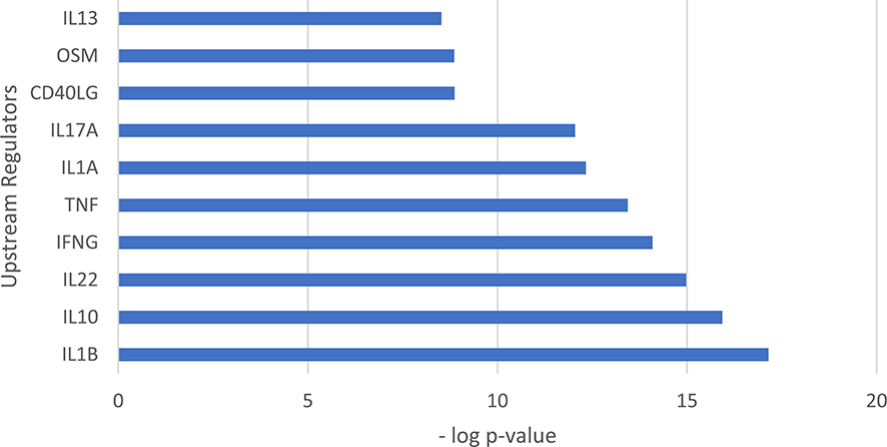
Upstream regulation of genes encoding antimicrobial peptides identified in LCs. Bar graph showing the top 10 upstream regulators ranked by p value. The top upstream regulator was *IL1B* (p= 7.07x10^-18^).

**Figure 5:**
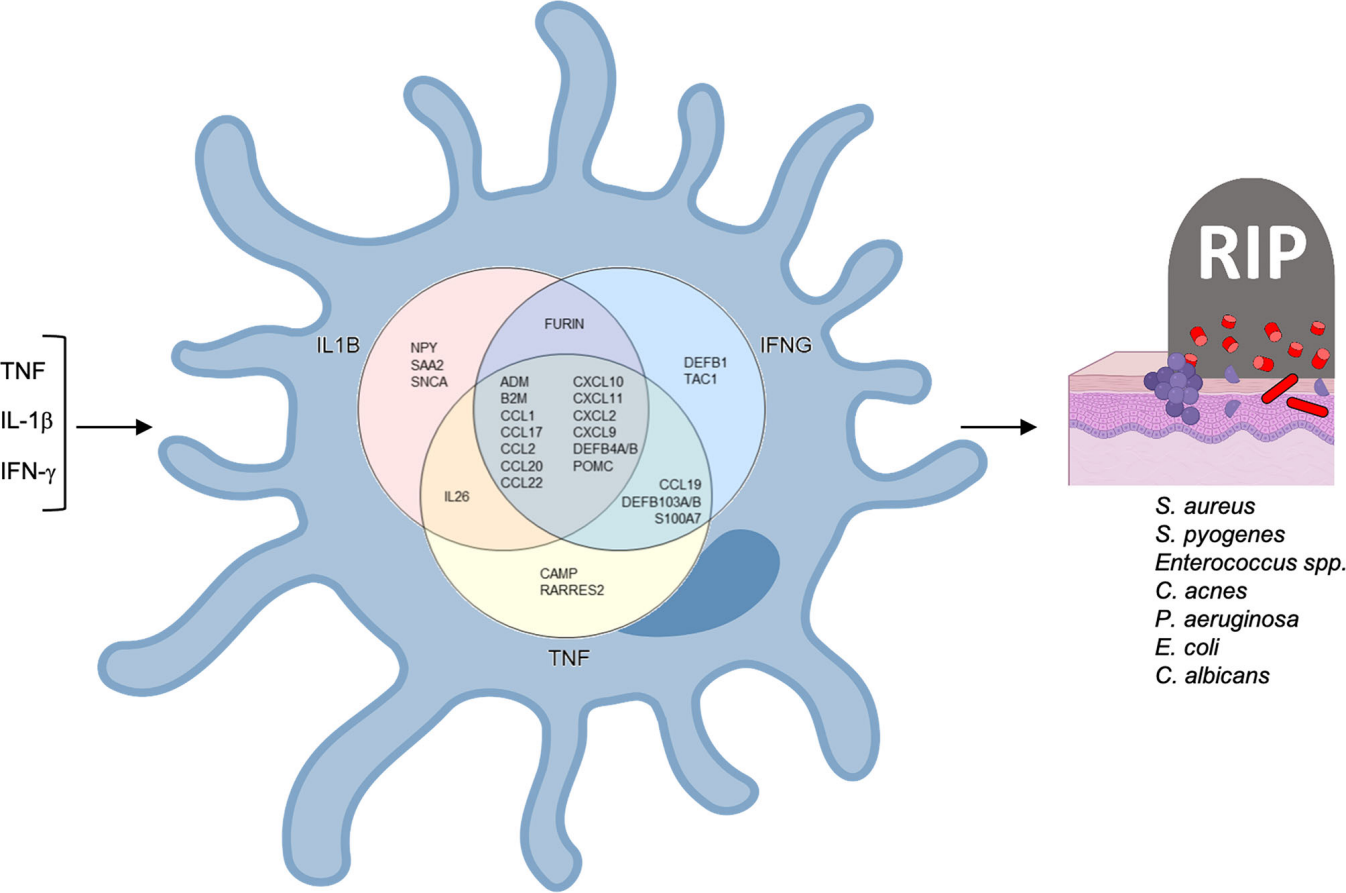
Antimicrobial network induced by *TNF, IFNG* and *IL1B* in LCs. *TNF* and *IFNG* were each identified as upstream regulators of 19 antimicrobial genes and *IL1B* as the upstream regulator of 18 antimicrobial genes.

We used Ingenuity Pathways Analysis and its knowledge database to identify the predicted upstream regulators of the 31 antimicrobial genes identified in LCs. Of the genes that encode cytokines, the top upstream regulator was *IL1B*, (p= 7.07x10^-18^) (**Figure 4**). The top 5 upstream regulator genes encode IL-1β, IFN-γ and TNF, all have been reported to induce one or more of the 30 LC antimicrobial genes *in vitro* (52). *TNF* was identified as the upstream regulator of 20 antimicrobial genes, followed by *IFNG* as the upstream regulator of 19 antimicrobial genes and *IL1B* as the upstream regulator of 18 antimicrobial genes. Together, the three cytokine genes were identified as upstream regulators for 25 of the 31 antimicrobial genes (**Figure 5**). In addition, we examined the target genes for other top upstream regulators that are known to contribute to the pathogenesis of skin disease: IL-10 (n=14 downstream genes), IL-22 (n=10), IL-13 (n=9), IL-17A (n=9). Thus, the antimicrobial gene response would likely be influenced by the local cytokine environment.

Of the 20 genes predicted to be induced by TNF, we detected nine genes, *ADM, CXCL2, CCL17, CCL27, IL26, CCL19, CCL2, CCL20* and *CCL1*, that were also upregulated in the transcriptomes of TNF-treated LCs. Of these, CCL17 protein has been validated to be induced by TNF *in vitro* (52). Although the Ingenuity pathways analysis did not predict TNF as an upstream regulator of *CXCL10*, TNF induced LCs to secrete CXCL10 *in vitro* (65).

Ingenuity pathways analysis identified IL1B as the upstream regulator for 18 of the 31 antimicrobial genes we identified in LC transcriptomes and/or the literature. For one these genes, the IL-1 family member, IL-1α, induces *CCL17* encoded peptide in LCs (52). was validated to induce CCL17 peptide (Alferink et al., 2003). The addition of IFN-γ to LCs leads to the induction of *CAMP* and *DEFB4* encoded peptides (33), as well as *CXCL9, CXCL10*, and *CXCL11* mRNAs (60).

Overall, we identified 31 antimicrobial genes in LCs, of which eight genes were induced by activation with TNF in transcriptomes, 16 additional genes by comparison of LCs to other cell types, of which all but one gene were unique, and eight additional genes were identified in LCs in publications. Of the 31 genes, 12 genes belonged to the chemokine superfamily and making it the largest family of antimicrobial genes identified in LCs. Additionally, according to the Antimicrobial Peptide Database (APD) (35), of the 31 antimicrobial genes identified in LCs, 29 encode proteins that are antibacterial. Of the 29 genes, 23 encode peptides with activity against gram-positive bacillus *Staphylococcus aureus*, which is the leading cause of skin and soft tissue infections (66–68) (**Supplementary Table S6**). A total of 18 of the 31 genes encode proteins that are antifungal, six are antiviral, and five are antiparasitic (**Supplementary Table S5**).

## Discussion

The localization of LCs to the epidermis provides a first line of defense for the innate immune system to defend the host against microbial pathogens invading the skin. Surprisingly, few pathways have been identified by which LCs mediate antimicrobial responses against viruses (31, 32), bacteria, and fungi (33). Here, in order to gain insight into the breadth of mechanisms by which LCs are equipped to mount an antimicrobial response, we searched publicly available databases for LC transcriptomes and also reviewed the literature to identify genes which encode proteins with direct antimicrobial activity against cutaneous pathogens. Overall, we identified 31 genes encoding proteins with direct antimicrobial activity, ten of which were identified in at least two different experiments, thus representing a core set of genes that comprise the LC antimicrobial gene program. Seven of these ten antimicrobial genes encode chemokines, *CCL1, CCL17, CCL19, CCL2, CCL22, CXCL14* and *CXCL2*, which mediate both antimicrobial and inflammatory responses. *CCL22* was identified in seven of nine transcriptomes in eight total comparisons, as well as validated in cultured LCs by PCR (51). As such, LCs are armed with an antimicrobial gene program to combat microbial pathogens.

Chemokines were the largest family of antimicrobial genes identified in LCs, accounting for 12 of the 31 genes, including seven of the ten genes that were cross-validated in at least two studies. Of the 12 genes, seven belonged to the chemokine family with a “CC” structure and five to the family with the “CXC” structure. Chemokines are pro-inflammatory, such that as part of host defense against microbial pathogens their trigger the migration of immune cells to the site of infection (69). However, many chemokines have a dual function, as they possess direct microbicidal activity (36, 37, 39, 70). Of the chemokines, *CCL22* was the most frequently detected antimicrobial gene, expressed in six different LC transcriptomes when compared to other cell types. *CCL22* was also previously identified in mature LCs cocultured with keratinocytes (51). CCL22 is one of the natural ligands for CCR4, along with CCL17 and CCL2. Both *CCL17* and *CCL22* were also upregulated in TNF treated LCs, with CCL17 protein induced in LCs by TNF *in vitro* (52). CCR4 is highly expressed by skin-infiltrating lymphocytes (71) and is involved in skin homing (72–74) of Th2 T cells, Th17 cells, Th22 cells and Tregs (75–79). LCs, by expression of *CCL22, CCL17*, and *CCL2* have the potential to recruit a range of functional CCR4^+^ T cell subpopulations to the site of disease.

Three of the top five upstream regulators of the 31 antimicrobial genes detected in LCs, *TNF*, *IL1B* and *IFNG*, have been corroborated by *in vitro* studies in which the cytokine was directly added to LCs. In the two data series in which TNF was added to activate migratory LCs *in vitro*, eight antimicrobial genes were identified (24, 49), all consistent with the TNF-downstream genes in the Ingenuity knowledge database. TNF is known to induce the maturation and migration of LCs (19, 80), increasing the number of LCs (65), and induce the expression of inflammatory genes in LCs (49, 65, 81).

Of the eight TNF inducible genes in migrating LCs, six encode chemokines, *CCL1, CCL2, CCL17, CCL19* and *CCL20*, which along with *IL26* were only detected in the transcriptomes of TNF activated LCs but not in LCs compared to transcriptomes of other myeloid cell types. Three of these antimicrobial genes have been corroborated in published papers; CCL1 protein has been identified in epidermal LCs *in situ* (54), CCL17 protein in IL-1α or TNF-activated LCs *in vivo* in mice (52) and *CXCL2* mRNA in freshly isolated murine LC cells (53). In addition to TNF, other inflammatory stimuli have been reported to induce the expression of genes in LCs encoding directly antimicrobial peptides. *CAMP* and *DEFB4* encoded peptides are induced in LCs by IFN-γ (33). *CXCL9, CXCL10*, and *CXCL11* mRNAs are induced in LCs by stimuli including IFN-γ, LPS, and poly I:C (32, 60). *NPY* mRNA expression in LCs is enhanced by GM-CSF and LPS (62). In addition, LCs have been shown to express *POMC* mRNA upon activation (61). Therefore, the activation and/or maturation of LCs triggers expression of multiple antimicrobial genes.

By comparing the expression of antimicrobial genes in LCs to other cell types, we identified 23 genes that arm LCs with the capacity to combat cutaneous pathogens and eight additional genes described in the literature to be expressed by LCs. Of these 31 genes, 23 genes encode peptides with activity against gram-positive bacillus *Staphylococcus aureus*, which is the leading cause of skin and soft tissue infections (66–68). LC expression of CAMP and DEFB4 results in an antimicrobial activity against the cutaneous pathogens including *M. leprae, S. aureus, Streptococcus pyogenes* and *Candida albicans* (33). In addition, LC have been previously shown to mediate an antiviral activity (32, 82, 83), although the mechanisms involved are not clear.

We previously found that the antimicrobial activity of LCs leads to killing and subsequent processing of microbial antigens facilitating antigen presentation to T cells (33). Some of the antimicrobial peptides expressed by migratory LCs have been shown to be pro-inflammatory, such as CCL22 and CCL17, which both act as a chemoattractant for CCR4-expressing T cells promoting LC:T cell interaction (84). Thus, the ability of LCs, in particular migratory LCs, to upregulate antimicrobial peptides links the innate and adaptive immune response, defending the host against cutaneous pathogens. There are at least two possible contributions of antimicrobial gene expression in migrating LCs.

We found that ten of the antimicrobial genes expressed in LCs were cross-validated by various methodologies, identifying a core set of genes by which LCs can contribute to host defense, that provide a basis for further functional studies. Any one antimicrobial gene may be sufficient to mediate an antimicrobial response, given our published data that IFN-γ upregulation of CAMP was required for antimicrobial activity in LCs (33). This was demonstrated by knockdown of the *CAMP* gene and the use of neutralizing monoclonal antibodies to IFN-γ (33). These strategies provide a strategy to determine whether the upregulation of multiple antimicrobial genes by cytokines and cell surface receptors such as Toll-like receptor ligands leads to a more potent antimicrobial response. It should be possible to identify key LC pathways that could be leveraged by immune therapy augmenting LC antimicrobial responses to combat cutaneous infection.

## Data Availability Statement

The original contributions presented in the study are included in the article/**Supplementary Material**. Further inquiries can be directed to the corresponding author.

## Author Contributions

Conceptualization: AO and RM. Data curation: AH and JR. Formal analysis: AO, RM, RT, FM, and EK. Funding acquisition: RM. Investigation: AO and RM. Methodology: AO and RM. Project administration: AO and RM. Resources: RM, FM, and MP. Software: FM and MP. Supervision: RM. Validation: EK. Visualization: AO and RM. Writing—original draft preparation: AO and RM. Writing—review and editing: AO, RM, AH, RT, FM, and BA. All authors contributed to the article and approved the submitted version.

## Funding

NIH grants R01 AI022553 (RLM, MP), R01 AR040312 (RLM, MP), R01 AR073252 (RLM, MP), R01 AR074302 (RLM, MP), NHMRCIdeas Grant APP1181482 (AH), 5R21EB024767-02 (EK), 1R01AR075959-01 (EK) and 1R01CA245277-01A1 (EK).

## Conflict of Interest

The authors declare that the research was conducted in the absence of any commercial or financial relationships that could be construed as a potential conflict of interest.

## Publisher’s Note

All claims expressed in this article are solely those of the authors and do not necessarily represent those of their affiliated organizations, or those of the publisher, the editors and the reviewers. Any product that may be evaluated in this article, or claim that may be made by its manufacturer, is not guaranteed or endorsed by the publisher.
