# Supplementary material for: Identification of Genes Encoding Antimicrobial Proteins in Langerhans Cells
Source: Front Immunol. 2021 Aug 26;12:695373. doi: 10.3389/fimmu.2021.695373 (PMC8426439; doi:10.3389/fimmu.2021.695373)
Supplement: Supplementary file 1 [file Table_1.docx]

**Supplemental Table S1. LC transcriptomes mined for genes that encode antimicrobial proteins.** For each study, the GSE accession number, the method by which the cells were isolated, their stimulation, the cell type comparisons with numbers of samples, the antimicrobial genes (AMGs) detected, detection platform and experiment types.

| **Study** | **Accession** | **Isolation** | **Stimulation** | **Analysis** | **AMGs** | **Platform/ Experiment Type** |
| --- | --- | --- | --- | --- | --- | --- |
| (Széles, Póliska et al. 2010) | [GSE23618](https://www.ncbi.nlm.nih.gov/geo/query/acc.cgi) | LCs and CD1a^+^ dermal DCs, were isolated from skin following enzymatic digestion.  Monocyte-derived DCs and CD1c^+^ DCs were isolated from blood | None | LCs (n=3) vs DDCs (n=3)  LCs (n=3) vs moDCs (n=3)  LCs (n=3) vs blood CD1c^+^ mDCs (n=3) | LCs vs CD1a^+^  dermal DCs: No direct AMGs  LCs vs moDCs: *B2M, CCL20 CCL27, CXCL14, DEFB1, S100A7, SNCA*  LCs vs blood CD1c^+^ mDCs: *ADM, CCL20,*  *CCL22, CCL27*  *CXCL14,*  *CXCL2,*  *DEFB1,*  *S100A7*  *SNCA* | Platform: Affymetrix Human Genome U133 Plus 2.0 Array  Experiment type: Expression profiling by array |
| (Hutter, Kauer et al. 2012) | [GSE35340](https://www.ncbi.nlm.nih.gov/geo/query/acc.cgi?acc=GSE35340) | LCs were isolated from skin following enzymatic digestion. mDCs and pDCs were isolated from peripheral blood | None | LCs (n=3) vs Blood CD1c+ mDCs (n=3)  LCs (n=3) vs pDCs (n=3) | LCs vs blood CD1c^+^ mDCs: *ADM, B2M, CCL22, CXCL14*  LCs vs pDCs: *CCL22, ADM, CXCL14, GAPDH* | Platform: Affymetrix Human Genome U133 Plus 2.0 Array  Experiment type: Expression profiling by array |
| (Boyd, Bennuru et al. 2013) | [GSE42694](https://www.ncbi.nlm.nih.gov/geo/query/acc.cgi?acc=GSE42694) | LCDCs were generated in vitro and exposed to live mosquito-derived third-stage larvae (L3) | Live mosquito-derived, third-stage larvae (L3) of *Brugia malayi* for 48h vs culture media | LCDCs exposed to live mosquito-derived, third-stage larvae (L3) (n=6) vs LCs (n =6) | No direct AMGs | Platform: Illumina HumanHT-12 V3.0 expression beadchip  Experiment type: Expression profiling by array |
| (Polak, Thirdborough et al. 2014) | [GSE49475](https://www.ncbi.nlm.nih.gov/geo/query/acc.cgi?acc=GSE49475) | LCs and CD11c^+^ dermal DCs were isolated from skin following migration | TNF at 0h, 2h, 8h, and 24h | LCs (n=6) vs CD11c^+^ dermal DCs (n=6) at 0h.  LCs stimulated with TNF at 2h, 8h, and 24h (n=3 each) vs LCs (n=6) prior to stimulation (0h) | LCs vs CD11c^+^ dermal DCs: *CCL22, RARRES2*  LCs after stimulation with TNF: *ADM, CCL1, CCL2, CCL17, CCL19* | Platform: Affymetrix Human Genome U219 Array  Experiment type: Expression profiling by array |
| (McGovern, Schlitzer et al. 2014) | [GSE60317](https://www.ncbi.nlm.nih.gov/geo/query/acc.cgi?acc=GSE60317) | LCs, CD14^+^ dermal DCs, and CD14^+^ dermal macrophages (MAC) were isolated from skin following migration | None | LCs (n=3) vs dermal CD14+ DCs (n=4),  LCs (n=3) vs MAC (n=4) | LCs vs CD14^+^ dermal DCs: No direct AMGs  LCs vs CD14^+^ MAC: *CCL22* | Platform: Illumina HumanWG-6 v3.0 expression beadchip  Experiment type: Expression profiling by array |
| (Artyomov, Munk et al. 2015) | [GSE66355](https://www.ncbi.nlm.nih.gov/geo/query/acc.cgi?acc=GSE66355) | LCs, CD141^+^, CD14^+^, and CD141^-^CD14^-^ dermal DCs were isolated after 48h migration from skin | None | LCs (n=6) vs. CD141^+^ dermal DCs (n=3)  LCs (n=6) vs CD14^+^ dermal DCs (n=4)  LCs (n=6) vs CD141^-^CD14^-^ dermal DCs (n=4) | LCs vs CD141^+^ dermal DCs*: B2M, FAM3A, HMGN2*  LCs vs CD14^+^ dermal DCs*:*  *B2M, CCL22*  LCs vs CD141^-^CD14^-^ dermal DCs: *HMGN2, TAC1* | Platform: Illumina HumanHT-12 V4.0 expression beadchip  Experiment type: Expression profiling by array |
| (Sirvent, Vallejo et al. 2020) | [GSE120386](https://www.ncbi.nlm.nih.gov/geo/query/acc.cgi?acc=GSE120386) | LCs were isolated by migration from skin after 48h | TNF at 0h, 2h, and 24h | LCs after stimulation with TNF at 2h and 24h (n =3 each) vs LCs prior to stimulation (n =3) | *ADM, CCL2, CCL19, CCL20, CXCL2, IL26* | Platform: Illumina HiSeq 2500/ Illumina NextSeq 500  Experiment type: Expression profiling by high throughput sequencing |
| (Lim, Milne et al. 2020) | [GSE122673](https://www.ncbi.nlm.nih.gov/geo/query/acc.cgi?acc=GSE122673) | LCs and macrophages (MAC) were isolated from skin and pDCs were isolated from spleen following enzymatic digestion | None | LCs (n=3) vs pDCS (n=3)  LCs (n=3) vs MAC (n=3) | LCs vs pDCs: *CCL22, FURIN, GAPDH*  LCs vs dermal MAC:  *FURIN* | Platform: Illumina HumanHT-12 V4.0 expression beadchip  Experiment type: Expression profiling by array |
| (Rhodes, Botting et al. 2020) | N/A | All cells were isolated from human abdominal skin by enzymatic digestion | None | LCs (n=3) vs dermal langerin^-^ cDC2 (n=3)  LCs (n=3) vs dermal langerin^+^ cDC2 (n=3)  LCs (n=3) vs CD14^+^ CD1c^-^monocyte-derived macrophages  (n=3)  LCs (n=3) vs CD14^+^CD1c^+^ monocyte-derived dendritic cells (n=3) | LCs vs dermal langerin^-^ cDC2: *CXCL14, SAA2*  LCs vs dermal langerin^+^ cDC2: *LEAP2*  LCs vs CD14^+^ CD1c^-^monocyte-derived macrophages:  *CCL22, CXCL14*  LCs vs monocyte- derived CD14^+^CD1c^+^ dendritic cells:  *CCL22, CXCL14 LEAP2* | Platform: Illumina HiSeq 2500  platform  Experiment type: Expression profiling by high throughput sequencing |

**Supplemental Table 2: Antimicrobial genes upregulated in LCs after stimulation with TNF.** A total of 8 antimicrobial genes were upregulated in LCs after activation with TNF in transcriptomes 4 and 7. *ADM*, *CCL19*, and *CCL2* were identified in both transcriptomes.

| **Genes upregulated in LCs after stimulation with TNF-α** | **Transcriptome 4 (GSE49475)** | **Transcriptome 7 (GSE120386)** |
| --- | --- | --- |
| *ADM* | ✓ | ✓ |
| *CCL1* | ✓ |  |
| *CCL17* | ✓ |  |
| *CCL19* | ✓ | ✓ |
| *CCL2* | ✓ | ✓ |
| *CCL20* |  | ✓ |
| *CXCL2* |  | ✓ |
| *IL26* |  | ✓ |

**Supplemental Table S3: Antimicrobial genes preferentially expressed in LCs vs other cell types.**

A summary of the comparisons that were made between LCs and different DC subtypes with the antimicrobial genes identified in each comparison.

|  | **Category of comparison** | **Subcategory** | **Detailed Comparisons** | **Genes encoding peptides with direct antimicrobial activity** |
| --- | --- | --- | --- | --- |
| Transcriptome 6 (GSE66355) | DC | dermal DC | LCs vs CD14^+^ dermal DC | *CCL22, B2M* |
| Transcriptome 6 (GSE66355) | DC | dermal DC | LCs vs CD141^+^ dermal DC | *B2M, FAM3A, HMGN2* |
| Transcriptome 6 (GSE66355) | DC | dermal DC | LCs vs CD141^-^CD14^-^ dermal DCs | *HMGN2, TAC1* |
| Transcriptome 4 (GSE49475) | DC | dermal DC | LCs vs dermal CD11c^+^ DCs (0h) | *CCL22, RARRES2* |
| Transcriptome 5 (GSE60317) | DC | dermal DC | LCs vs dermal CD14^+^ DC |  |
| Transcriptome 1 (GSE23618) | DC | dermal DC | LCs vs dermal CD1a^+^ DCs |  |
| Transcriptome 9 | DC | dermal DC | LCs vs dermal langerin^-^ cDC2 | *CXCL14, SAA2* |
| Transcriptome 9 | DC | dermal DC | LCs vs dermal langerin^+^ cDC2 | *LEAP2* |
| Transcriptome 1 (GSE23618) | DC | mDC | LCs vs blood CD1c^+^ mDC | *ADM, CCL20, CCL22, CCL27, CXCL14, CXCL2, DEFB1,*  *S100A7, SNCA* |
| Transcriptome 2 (GSE35340) | DC | mDC | LCs vs blood CD1c^+^ mDC | *ADM, CXCL14, B2M* |
| Transcriptome 9 | DC | moDC | LCs vs CD14^+^ CD1c^+^ monocyte-derived DC | *CCL22, LEAP2* |
| Transcriptome 1 (GSE23618) | DC | moDC | LCs vs monocyte-derived DC | *B2M, CCL20 CCL27, CXCL14, DEFB1, S100A7, SNCA* |
| Transcriptome 2 (GSE35340) | DC | pDC | LCs vs pDCs | *CCL22, ADM, CXCL14, GAPDH* |
| Transcriptome 8 (GSE122673) | DC | pDC | LCs vs pDCs | *CCL22, GAPDH, FURIN* |
| Transcriptome 5 (GSE60317) | macrophage | macrophage | LCs vs dermal CD14^+^ macrophages | *CCL22* |
| Transcriptome 8 (GSE122673) | macrophage | macrophage | LCs vs dermal macrophages | *FURIN* |
| Transcriptome 9 | macrophage | MDM | LCs vs CD14^+^ CD1c^-^ (monocyte derived macrophages) | *CCL22, CXCL14* |

**Supplemental Table S4: Antimicrobial genes in LCs: Literature Findings.**

A table of genes encoding antimicrobial peptides previously identified in LCs. *CCL1*, *CCL17*, *CCL22*, and *CXCL2* were also identified by mining the transcriptomes.

| **Genes encoding antimicrobial proteins identified in literature** | **Findings** |
| --- | --- |
| *CAMP* | *CAMP*-encoded peptide is induced in epidermal CD1a^+^ LCs by IFN-γ (Dang, Teles et al. 2019) |
| *CCL1* | *CCL1*-encoded peptide is expressed by epidermal LCs in situ (Schaerli, Ebert et al. 2004) |
| *CCL17* | LCs produce *CCL17*-encoded peptide after treatment with IL-1α or TNF (Alferink, Lieberam et al. 2003) |
| *CCL22* | *CCL22* mRNA is expressed in murine LCs during maturation (Ross, Ross et al. 1999) |
| *CXCL2* | *CXCL2* mRNA is expressed in freshly isolated LCs (Heufler, Topar et al. 1992) |
| *CXCL9* | *CXCL9*-encoded peptide is induced in LCs by stimuli including IFN-γ, LPS, and poly I:C (Fujita, Asahina et al. 2005) and in LCDCs after viral infection (Renn, Sanchez et al. 2006) |
| *CXCL10* | *CXCL10*-encoded peptide is induced in LCs by stimuli including IFN-γ, LPS, and poly I:C (Fujita, Asahina et al. 2005) and TNF (Berthier-Vergnes, Bermond et al. 2005) |
| *CXCL11* | *CXCL11*-encoded peptide is induced in LCs by stimuli including IFN-γ, LPS, and poly I:C(Fujita, Asahina et al. 2005) and in LCDCs after viral infection (Renn, Sanchez et al. 2006) |
| *DEFB4* | *DEFB4*-encoded peptide is induced in epidermal CD1a^+^ LCs by IFN-γ (Dang, Teles et al. 2019) |
| *DEFB103-*encoded peptide (HBD-3) | HBD-3 protein expression colocalizes with LCs in the epidermis (Pilkington, Dearman et al. 2018) and it is constitutively expressed in LCs in gingival epithelium in samples of patients with chronic periodontitis and in healthy subjects (Lu, Samaranayake et al. 2005) |
| *NPY* | The expression of *NPY* mRNA is enhanced in LCs by GM-CSF and LPS (Lambert, Campton et al. 2002) |
| *POMC* | LCs express *POMC* mRNA and release active α-MSH upon activation (Luger, Scholzen et al. 1997) |

**Supplemental Table S5: Summary of genes encoding antimicrobial peptides in LCs as well as their antimicrobial activities.** 15 genes were identified from analysis of transcriptomes and 8 additional genes were identified in the literature in non-transcriptome studies. 29 encode proteins that are antibacterial, 18 are antifungal, six are antiviral, and five are antiparasitic**.**

**Supplemental Table S6: Genes encoding antimicrobial peptides with activity against *Staphylococcus Aureus*.** 23 of the 30 genes possess antibacterial activity against the gram-positive bacterium *Staphylococcus aureus*, which is the main causative agent of skin and soft tissue infections.

| ***Genes*** | ***References*** |
| --- | --- |
| *ADM* | (Allaker, Zihni et al. 1999) |
| *B2M* | (Kim, Park et al. 2012) |
| *CAMP* | (Agerberth, Gunne et al. 1995) |
| *CCL1* | (Yang, Chen et al. 2003) |
| *CCL17* |  |
| *CCL20* |  |
| *CCL22* |  |
| *CXCL2* |  |
| *CXCL10* |  |
| *CXCL9* |  |
| *CXCL11* |  |
| *CXCL14* | (Maerki, Meuter et al. 2009) |
| *DEFB1* | (Raschig, Mailänder-Sánchez et al. 2017) |
| *DEFB4* | (Harder, Bartels et al. 1997) |
| *DEFB103A* | (Sass, Schneider et al. 2010) |
| *FURIN* | (Sinha, Harioudh et al. 2018) |
| *IL26* | (Meller, Di Domizio et al. 2015) |
| *LEAP2* | (Krause, Sillard et al. 2003) |
| *POMC* | (Cutuli, Cristiani et al. 2000) |
| *RARRES2* | (Banas, Zabieglo et al. 2013) |
| *SAA2* | (Zheng, Li et al. 2020) |
| *SNCA* | (Park, Moon et al. 2016) |
| *TAC1* | (Kowalska, Carr et al. 2002) |

**Supplementary Table S7. Canonical pathways in digested LCs.** The canonical pathways in LCs vs pDCs, CD1+mDCs and CD14+ dermal DCs were calculated using Ingenuity Pathways Analysis. For each comparison, the data were sorted according to the -log(BH) p values, choosing those pathways that appeared in 5/6 or 6/6 comparisons. The pathways were then mined for genes that had positive -log(BH) p values in at least 4/6 comparisons.

**Supplemental Fig. S1. *B2M* expression in LCs vs other DC subtypes.** B2M was preferentially expressed in LCs vs other DC subtypes in a total of 3 instances.
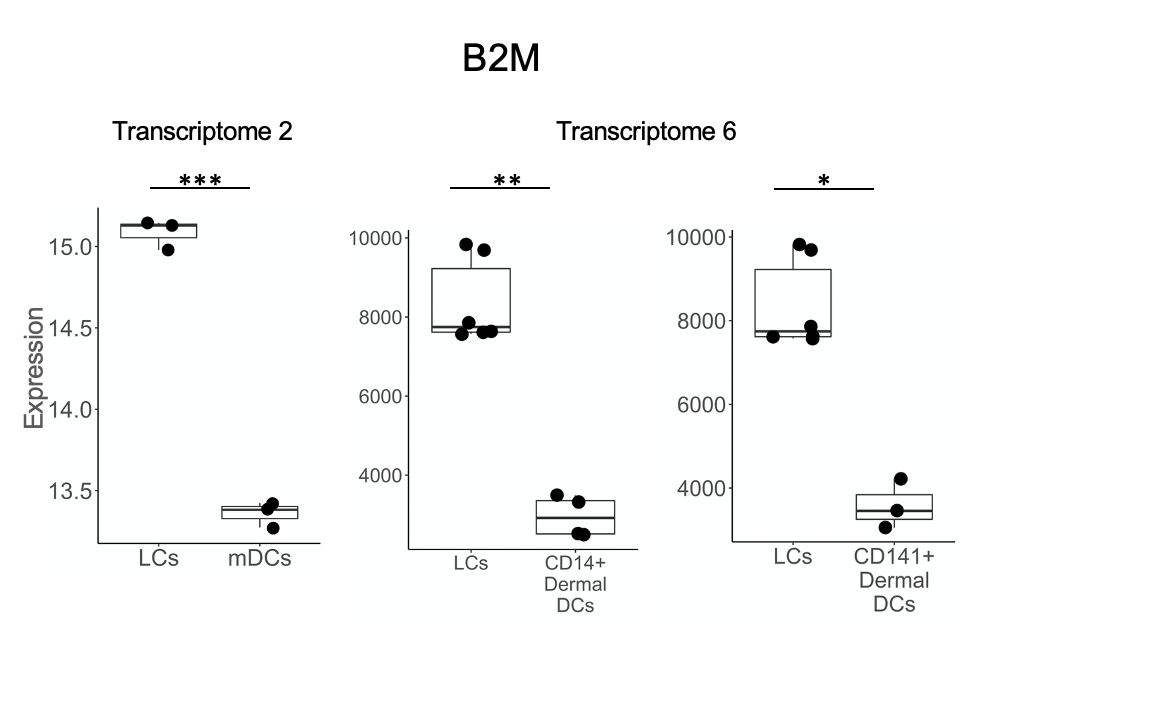


**Supplemental Fig. S2. *GAPDH* expression in LCs vs other DC subtypes.** *GADPH* was preferentially expressed in LCs vs other DC subtypes in two different instances in LCs vs pDCs.

**
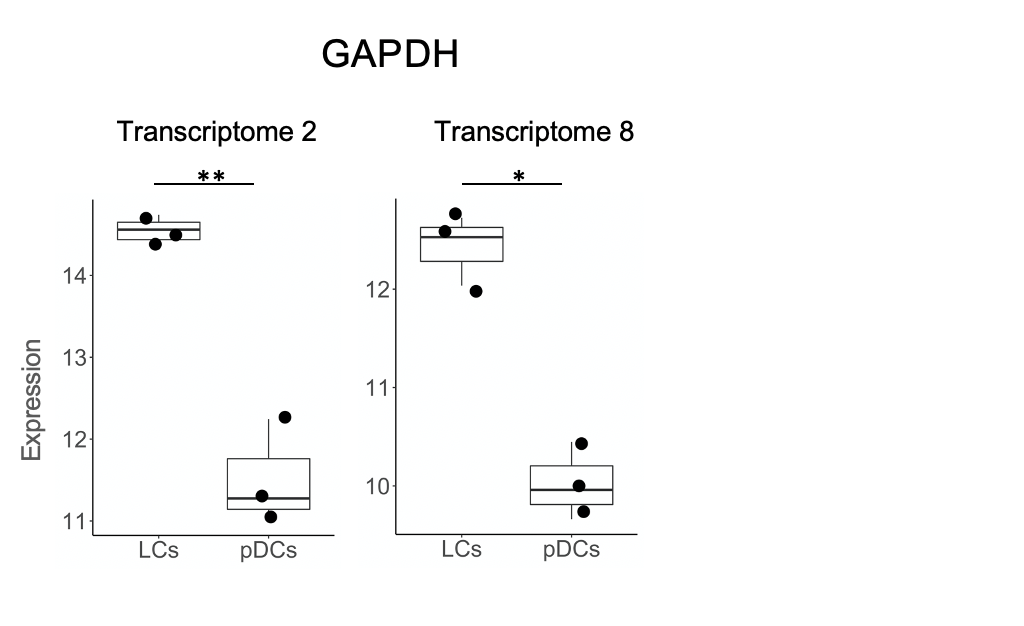
**

**Supplemental Fig. S3. *HMGN2* expression in LCs vs other DC subtypes.** *HMGN2* was preferentially expressed in LCs in two instances in transcriptome 6.

**
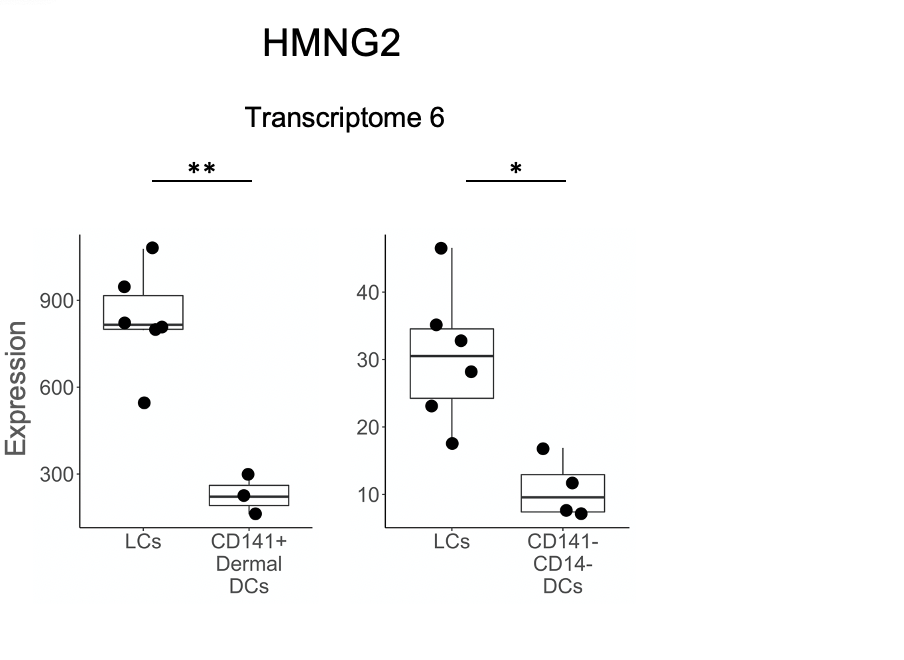
**

**Supplemental Fig. S4. Heat map of genes preferentially expressed in three of more comparisons**. We created heatmaps using the expression value provided by GEO2R for each gene. Each individual dataset was normalized using the conditional formatting tool on Microsoft Excel on a scale from 0 being white to red being the maximal amount. Each box represents an individual sample from each dataset. Six of the 23 genes identified in the transcriptomes have been identified in 3 or more different comparisons. *CCL22* was found to be expressed at higher levels in LCs vs pDCs across 2 different transcriptomes (T2 and T8). *CXCL14* was be expressed at higher levels in LCs vs blood CD1c^+^ DCs in 2 different transcriptomes (T1 and T2). *ADM* was found to be higher expressed in LCs after 2h stimulation with TNF than LCs prior to TNF stimulation (0 hours). *CCL19* was upregulated in LCs treated with TNF for 24 hours vs 0 hours (T4 and T7).


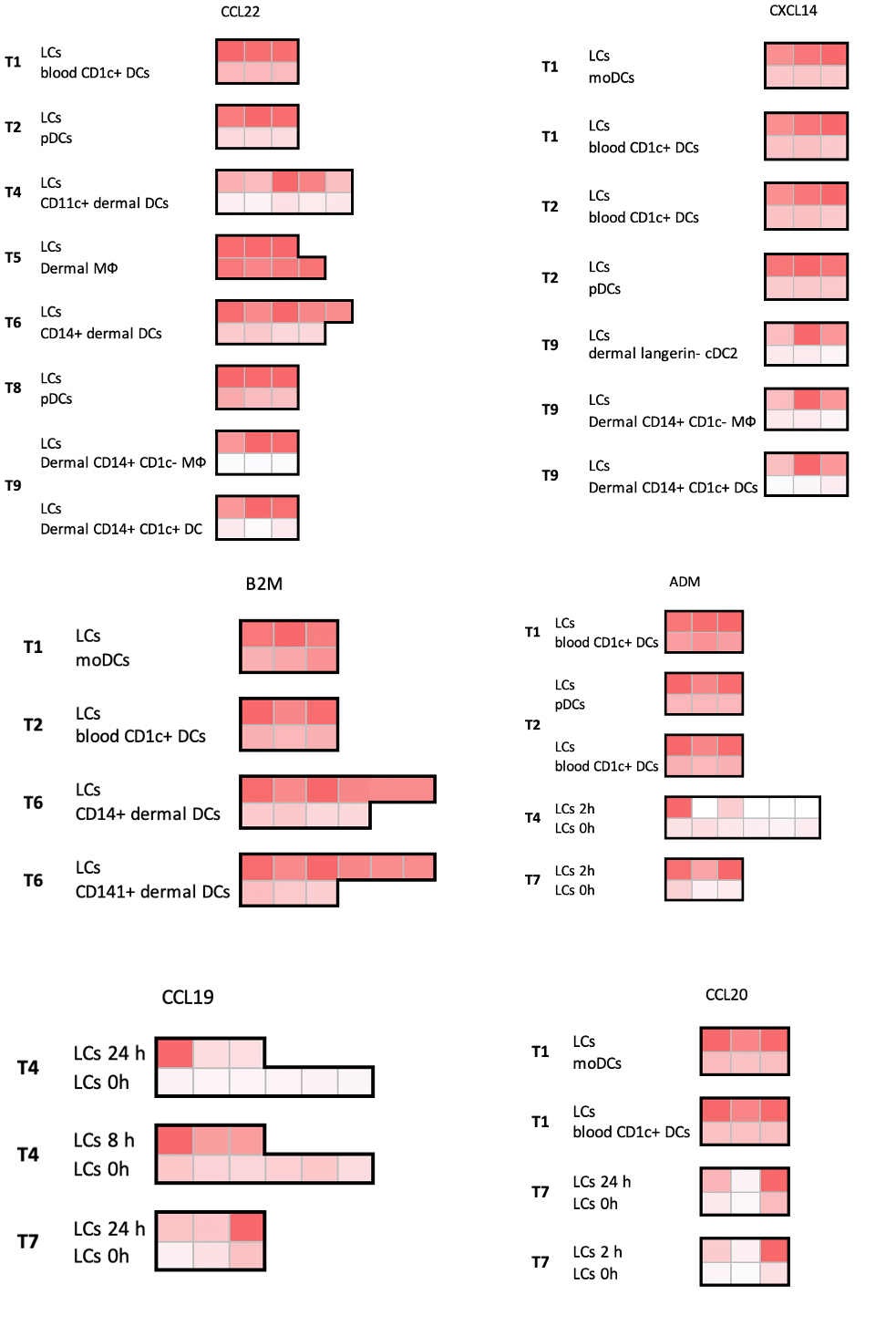


**Supplemental Fig. S5. Heat map of genes preferentially expressed in two comparisons.** *GAPDH* was found to be more strongly expressed in LCs vs pDCs in Transcriptomes 2 and 8. *CCL2* was upregulated in LCs treated with TNF for 24 hours vs 0 hours in T4 and T7.


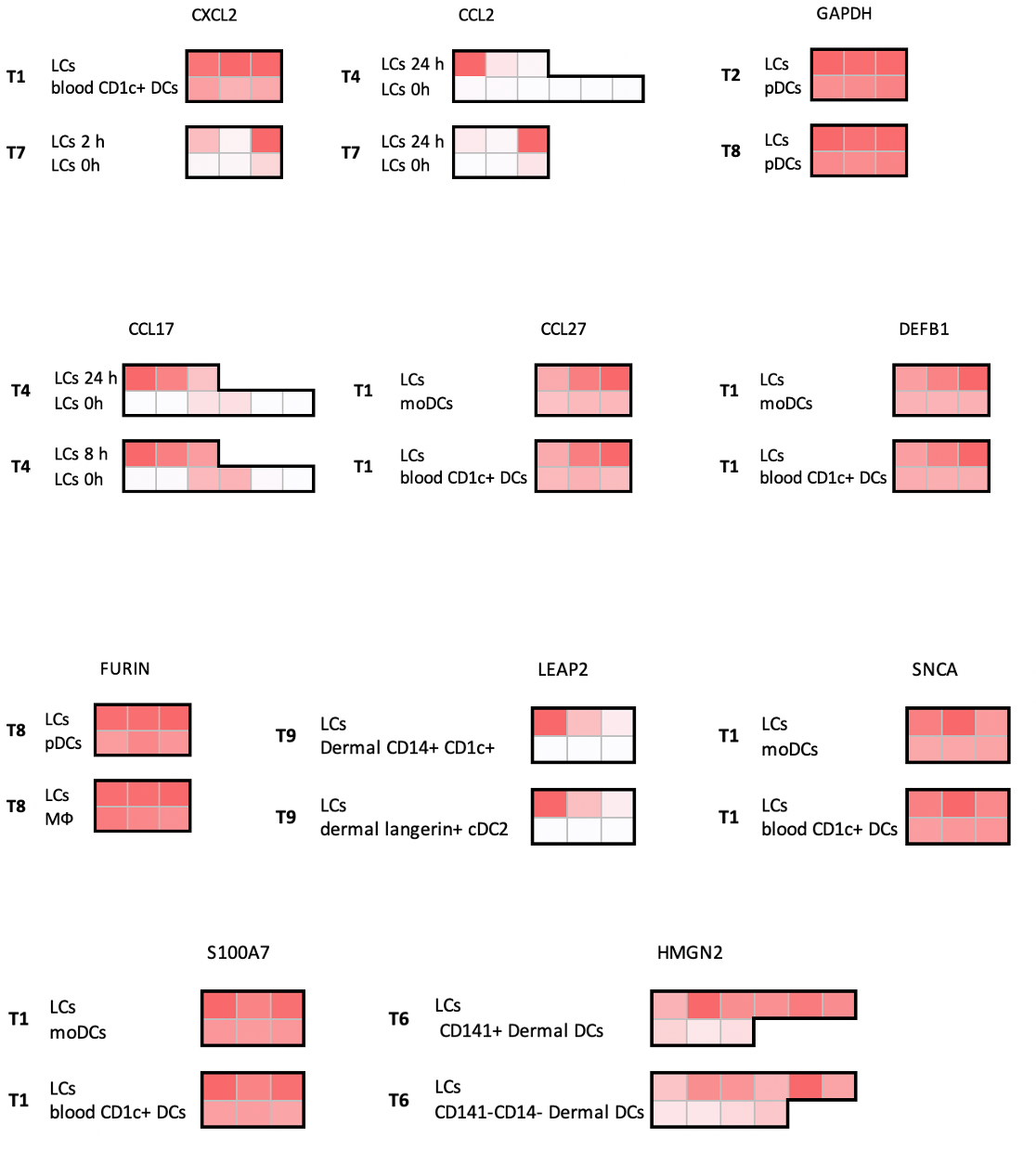
**Supplemental Fig. S6. Heat map of genes preferentially expressed in one comparison.** 4 of the 23 genes identified in transcriptomes were only found in one instance each.


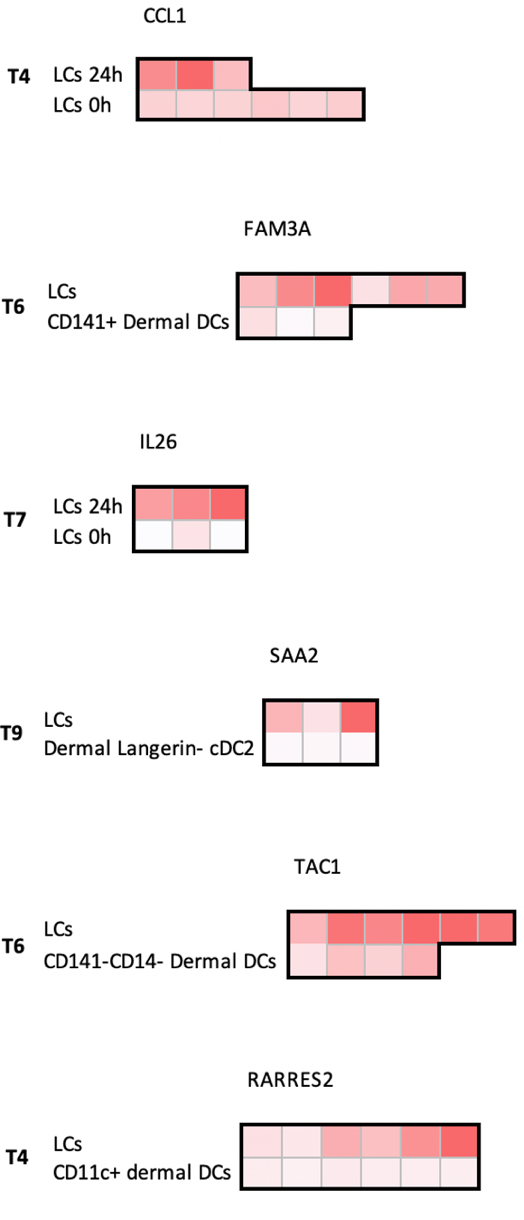


**REFERENCES**

Agerberth, B., H. Gunne, J. Odeberg, P. Kogner, H. G. Boman and G. H. Gudmundsson (1995). "FALL-39, a putative human peptide antibiotic, is cysteine-free and expressed in bone marrow and testis." Proceedings of the National Academy of Sciences of the United States of America 92(1): 195-199.

Alferink, J., I. Lieberam, W. Reindl, A. Behrens, S. Weiss, N. Hüser, K. Gerauer, R. Ross, A. B. Reske-Kunz, P. Ahmad-Nejad, H. Wagner and I. Förster (2003). "Compartmentalized production of CCL17 in vivo: strong inducibility in peripheral dendritic cells contrasts selective absence from the spleen." J Exp Med 197(5): 585-599.

Allaker, R. P., C. Zihni and S. Kapas (1999). "An investigation into the antimicrobial effects of adrenomedullin on members of the skin, oral, respiratory tract and gut microflora." FEMS Immunol Med Microbiol 23(4): 289-293.

Artyomov, M. N., A. Munk, L. Gorvel, D. Korenfeld, M. Cella, T. Tung and E. Klechevsky (2015). "Modular expression analysis reveals functional conservation between human Langerhans cells and mouse cross-priming dendritic cells." J Exp Med 212(5): 743-757.

Banas, M., K. Zabieglo, G. Kasetty, M. Kapinska-Mrowiecka, J. Borowczyk, J. Drukala, K. Murzyn, B. A. Zabel, E. C. Butcher, J. M. Schroeder, A. Schmidtchen and J. Cichy (2013). "Chemerin is an antimicrobial agent in human epidermis." PloS one 8(3): e58709-e58709.

Berthier-Vergnes, O., F. Bermond, V. Flacher, C. Massacrier, D. Schmitt and J. Péguet-Navarro (2005). "TNF-alpha enhances phenotypic and functional maturation of human epidermal Langerhans cells and induces IL-12 p40 and IP-10/CXCL-10 production." FEBS Lett 579(17): 3660-3668.

Boyd, A., S. Bennuru, Y. Wang, V. Sanprasert, M. Law, D. Chaussabel, T. B. Nutman and R. T. Semnani (2013). "Quiescent innate response to infective filariae by human Langerhans cells suggests a strategy of immune evasion." Infect Immun 81(5): 1420-1429.

Cole, K. E., C. A. Strick, T. J. Paradis, K. T. Ogborne, M. Loetscher, R. P. Gladue, W. Lin, J. G. Boyd, B. Moser, D. E. Wood, B. G. Sahagan and K. Neote (1998). "Interferon-inducible T cell alpha chemoattractant (I-TAC): a novel non-ELR CXC chemokine with potent activity on activated T cells through selective high affinity binding to CXCR3." The Journal of experimental medicine 187(12): 2009-2021.

Cutuli, M., S. Cristiani, J. M. Lipton and A. Catania (2000). "Antimicrobial effects of alpha-MSH peptides." J Leukoc Biol 67(2): 233-239.

Dang, A. T., R. M. Teles, P. T. Liu, A. Choi, A. Legaspi, E. N. Sarno, M. T. Ochoa, K. Parvatiyar, G. Cheng, M. Gilliet, B. R. Bloom and R. L. Modlin (2019). "Autophagy links antimicrobial activity with antigen presentation in Langerhans cells." JCI insight 4(8): e126955.

Fujita, H., A. Asahina, M. Sugaya, K. Nakamura, P. Gao, H. Fujiwara and K. Tamaki (2005). "Differential production of Th1- and Th2-type chemokines by mouse Langerhans cells and splenic dendritic cells." J Invest Dermatol 124(2): 343-350.

Harder, J., J. Bartels, E. Christophers and J. M. Schröder (1997). "A peptide antibiotic from human skin." Nature 387(6636): 861.

Heufler, C., G. Topar, F. Koch, B. Trockenbacher, E. Kämpgen, N. Romani and G. Schuler (1992). "Cytokine gene expression in murine epidermal cell suspensions: interleukin 1 beta and macrophage inflammatory protein 1 alpha are selectively expressed in Langerhans cells but are differentially regulated in culture." J Exp Med 176(4): 1221-1226.

Hutter, C., M. Kauer, I. Simonitsch-Klupp, G. Jug, R. Schwentner, J. Leitner, P. Bock, P. Steinberger, W. Bauer, N. Carlesso, M. Minkov, H. Gadner, G. Stingl, H. Kovar and E. Kriehuber (2012). "Notch is active in Langerhans cell histiocytosis and confers pathognomonic features on dendritic cells." Blood 120(26): 5199-5208.

Kim, J. Y., S. C. Park, J. K. Lee, S. J. Choi, K. S. Hahm and Y. Park (2012). "Novel antibacterial activity of β(2)-microglobulin in human amniotic fluid." PLoS One 7(11): e47642.

Kowalska, K., D. B. Carr and A. W. Lipkowski (2002). "Direct antimicrobial properties of substance P." Life Sci 71(7): 747-750.

Krause, A., R. Sillard, B. Kleemeier, E. Klüver, E. Maronde, J. R. Conejo-García, W. G. Forssmann, P. Schulz-Knappe, M. C. Nehls, F. Wattler, S. Wattler and K. Adermann (2003). "Isolation and biochemical characterization of LEAP-2, a novel blood peptide expressed in the liver." Protein Sci 12(1): 143-152.

Lambert, R. W., K. Campton, W. Ding, H. Ozawa and R. D. Granstein (2002). "Langerhans cell expression of neuropeptide Y and peptide YY." Neuropeptides 36(4): 246-251.

Lim, K. P. H., P. Milne, M. Poidinger, K. Duan, H. Lin, N. McGovern, H. Abhyankar, D. Zinn, T. M. Burke, O. S. Eckstein, R. Chakraborty, A. Sengal, B. Scull, E. Newell, M. Merad, K. L. McClain, T. K. Man, F. Ginhoux, M. Collin and C. E. Allen (2020). "Circulating CD1c+ myeloid dendritic cells are potential precursors to LCH lesion CD1a+CD207+ cells." Blood Adv 4(1): 87-99.

Lu, Q., L. P. Samaranayake, R. P. Darveau and L. Jin (2005). "Expression of human beta-defensin-3 in gingival epithelia." J Periodontal Res 40(6): 474-481.

Luger, T. A., T. Scholzen and S. Grabbe (1997). "The role of alpha-melanocyte-stimulating hormone in cutaneous biology." J Investig Dermatol Symp Proc 2(1): 87-93.

Maerki, C., S. Meuter, M. Liebi, K. Mühlemann, M. J. Frederick, N. Yawalkar, B. Moser and M. Wolf (2009). "Potent and broad-spectrum antimicrobial activity of CXCL14 suggests an immediate role in skin infections." J Immunol 182(1): 507-514.

McGovern, N., A. Schlitzer, M. Gunawan, L. Jardine, A. Shin, E. Poyner, K. Green, R. Dickinson, X. N. Wang, D. Low, K. Best, S. Covins, P. Milne, S. Pagan, K. Aljefri, M. Windebank, D. Miranda-Saavedra, A. Larbi, P. S. Wasan, K. Duan, M. Poidinger, V. Bigley, F. Ginhoux, M. Collin and M. Haniffa (2014). "Human dermal CD14⁺ cells are a transient population of monocyte-derived macrophages." Immunity 41(3): 465-477.

Meller, S., J. Di Domizio, K. S. Voo, H. C. Friedrich, G. Chamilos, D. Ganguly, C. Conrad, J. Gregorio, D. Le Roy, T. Roger, J. E. Ladbury, B. Homey, S. Watowich, R. L. Modlin, D. P. Kontoyiannis, Y. J. Liu, S. T. Arold and M. Gilliet (2015). "T(H)17 cells promote microbial killing and innate immune sensing of DNA via interleukin 26." Nat Immunol 16(9): 970-979.

Park, S. C., J. C. Moon, S. Y. Shin, H. Son, Y. J. Jung, N. H. Kim, Y. M. Kim, M. K. Jang and J. R. Lee (2016). "Functional characterization of alpha-synuclein protein with antimicrobial activity." Biochem Biophys Res Commun 478(2): 924-928.

Pilkington, S. M., R. J. Dearman, I. Kimber and C. E. M. Griffiths (2018). "Langerhans cells express human β-defensin 3: relevance for immunity during skin ageing." Br J Dermatol 179(5): 1170-1171.

Polak, M. E., S. M. Thirdborough, C. Y. Ung, T. Elliott, E. Healy, T. C. Freeman and M. R. Ardern-Jones (2014). "Distinct molecular signature of human skin Langerhans cells denotes critical differences in cutaneous dendritic cell immune regulation." J Invest Dermatol 134(3): 695-703.

Raschig, J., D. Mailänder-Sánchez, A. Berscheid, J. Berger, A. A. Strömstedt, L. F. Courth, N. P. Malek, H. Brötz-Oesterhelt and J. Wehkamp (2017). "Ubiquitously expressed Human Beta Defensin 1 (hBD1) forms bacteria-entrapping nets in a redox dependent mode of action." PLoS Pathog 13(3): e1006261.

Renn, C. N., D. J. Sanchez, M. T. Ochoa, A. J. Legaspi, C. K. Oh, P. T. Liu, S. R. Krutzik, P. A. Sieling, G. Cheng and R. L. Modlin (2006). "TLR activation of Langerhans cell-like dendritic cells triggers an antiviral immune response." J Immunol 177(1): 298-305.

Rhodes, J. W., R. A. Botting, K. M. Bertram, H. Rana, H. Baharlou, E. E. Longmuir-Vine, P. Vegh, J. Fletcher, T. R. O’Neil, G. P. Parnell, J. D. Graham, N. Nasr, J. J. K. Lim, L. Barnouti, P. Haertsch, M. P. Gosselink, A. Di Re, G. Ctercteko, G. J. Jenkins, A. J. Brooks, E. Patrick, S. N. Byrne, M. A. Haniffa, A. L. Cunningham and A. N. Harman (2020). "Identification of HIV-Transmitting Sub-Epithelial Mononuclear Phagocytes in Human Anogenital and Colorectal Tissues." bioRxiv: 2020.2005.2026.117408.

Ross, R., X. L. Ross, H. Ghadially, T. Lahr, J. Schwing, J. Knop and A. B. Reske-Kunz (1999). "Mouse langerhans cells differentially express an activated T cell-attracting CC chemokine." J Invest Dermatol 113(6): 991-998.

Sass, V., T. Schneider, M. Wilmes, C. Körner, A. Tossi, N. Novikova, O. Shamova and H. G. Sahl (2010). "Human beta-defensin 3 inhibits cell wall biosynthesis in Staphylococci." Infect Immun 78(6): 2793-2800.

Schaerli, P., L. Ebert, K. Willimann, A. Blaser, R. S. Roos, P. Loetscher and B. Moser (2004). "A skin-selective homing mechanism for human immune surveillance T cells." J Exp Med 199(9): 1265-1275.

Sinha, S., M. K. Harioudh, R. P. Dewangan, W. J. Ng, J. K. Ghosh and S. Bhattacharjya (2018). "Cell-Selective Pore Forming Antimicrobial Peptides of the Prodomain of Human Furin: A Conserved Aromatic/Cationic Sequence Mapping, Membrane Disruption, and Atomic-Resolution Structure and Dynamics." ACS Omega 3(11): 14650-14664.

Sirvent, S., A. F. Vallejo, J. Davies, K. Clayton, Z. Wu, J. Woo, J. Riddell, V. K. Chaudhri, P. Stumpf, L. A. Nazlamova, G. Wheway, M. Rose-Zerilli, J. West, M. Pujato, X. Chen, C. H. Woelk, B. MacArthur, M. Ardern-Jones, P. S. Friedmann, M. T. Weirauch, H. Singh and M. E. Polak (2020). "Genomic programming of IRF4-expressing human Langerhans cells." Nat Commun 11(1): 313.

Széles, L., S. Póliska, G. Nagy, I. Szatmari, A. Szanto, A. Pap, M. Lindstedt, S. J. Santegoets, R. Rühl, B. Dezsö and L. Nagy (2010). "Research resource: transcriptome profiling of genes regulated by RXR and its permissive and nonpermissive partners in differentiating monocyte-derived dendritic cells." Mol Endocrinol 24(11): 2218-2231.

Yang, D., Q. Chen, D. M. Hoover, P. Staley, K. D. Tucker, J. Lubkowski and J. J. Oppenheim (2003). "Many chemokines including CCL20/MIP-3alpha display antimicrobial activity." J Leukoc Biol 74(3): 448-455.

Zheng, H., H. Li, J. Zhang, H. Fan, L. Jia, W. Ma, S. Ma, S. Wang, H. You, Z. Yin and X. Li (2020). "Serum amyloid A exhibits pH dependent antibacterial action and contributes to host defense against Staphylococcus aureus cutaneous infection." J Biol Chem 295(9): 2570-2581.

Agerberth, B., H. Gunne, J. Odeberg, P. Kogner, H. G. Boman and G. H. Gudmundsson (1995). "FALL-39, a putative human peptide antibiotic, is cysteine-free and expressed in bone marrow and testis." Proceedings of the National Academy of Sciences of the United States of America **92**(1): 195-199.

Alferink, J., I. Lieberam, W. Reindl, A. Behrens, S. Weiss, N. Hüser, K. Gerauer, R. Ross, A. B. Reske-Kunz, P. Ahmad-Nejad, H. Wagner and I. Förster (2003). "Compartmentalized production of CCL17 in vivo: strong inducibility in peripheral dendritic cells contrasts selective absence from the spleen." J Exp Med **197**(5): 585-599.

Allaker, R. P., C. Zihni and S. Kapas (1999). "An investigation into the antimicrobial effects of adrenomedullin on members of the skin, oral, respiratory tract and gut microflora." FEMS Immunol Med Microbiol **23**(4): 289-293.

Artyomov, M. N., A. Munk, L. Gorvel, D. Korenfeld, M. Cella, T. Tung and E. Klechevsky (2015). "Modular expression analysis reveals functional conservation between human Langerhans cells and mouse cross-priming dendritic cells." J Exp Med **212**(5): 743-757.

Banas, M., K. Zabieglo, G. Kasetty, M. Kapinska-Mrowiecka, J. Borowczyk, J. Drukala, K. Murzyn, B. A. Zabel, E. C. Butcher, J. M. Schroeder, A. Schmidtchen and J. Cichy (2013). "Chemerin is an antimicrobial agent in human epidermis." PloS one **8**(3): e58709-e58709.

Berthier-Vergnes, O., F. Bermond, V. Flacher, C. Massacrier, D. Schmitt and J. Péguet-Navarro (2005). "TNF-alpha enhances phenotypic and functional maturation of human epidermal Langerhans cells and induces IL-12 p40 and IP-10/CXCL-10 production." FEBS Lett **579**(17): 3660-3668.

Boyd, A., S. Bennuru, Y. Wang, V. Sanprasert, M. Law, D. Chaussabel, T. B. Nutman and R. T. Semnani (2013). "Quiescent innate response to infective filariae by human Langerhans cells suggests a strategy of immune evasion." Infect Immun **81**(5): 1420-1429.

Cutuli, M., S. Cristiani, J. M. Lipton and A. Catania (2000). "Antimicrobial effects of alpha-MSH peptides." J Leukoc Biol **67**(2): 233-239.

Dang, A. T., R. M. Teles, P. T. Liu, A. Choi, A. Legaspi, E. N. Sarno, M. T. Ochoa, K. Parvatiyar, G. Cheng, M. Gilliet, B. R. Bloom and R. L. Modlin (2019). "Autophagy links antimicrobial activity with antigen presentation in Langerhans cells." JCI insight **4**(8): e126955.

Fujita, H., A. Asahina, M. Sugaya, K. Nakamura, P. Gao, H. Fujiwara and K. Tamaki (2005). "Differential production of Th1- and Th2-type chemokines by mouse Langerhans cells and splenic dendritic cells." J Invest Dermatol **124**(2): 343-350.

Harder, J., J. Bartels, E. Christophers and J. M. Schröder (1997). "A peptide antibiotic from human skin." Nature **387**(6636): 861.

Heufler, C., G. Topar, F. Koch, B. Trockenbacher, E. Kämpgen, N. Romani and G. Schuler (1992). "Cytokine gene expression in murine epidermal cell suspensions: interleukin 1 beta and macrophage inflammatory protein 1 alpha are selectively expressed in Langerhans cells but are differentially regulated in culture." J Exp Med **176**(4): 1221-1226.

Hutter, C., M. Kauer, I. Simonitsch-Klupp, G. Jug, R. Schwentner, J. Leitner, P. Bock, P. Steinberger, W. Bauer, N. Carlesso, M. Minkov, H. Gadner, G. Stingl, H. Kovar and E. Kriehuber (2012). "Notch is active in Langerhans cell histiocytosis and confers pathognomonic features on dendritic cells." Blood **120**(26): 5199-5208.

Kim, J. Y., S. C. Park, J. K. Lee, S. J. Choi, K. S. Hahm and Y. Park (2012). "Novel antibacterial activity of β(2)-microglobulin in human amniotic fluid." PLoS One **7**(11): e47642.

Kowalska, K., D. B. Carr and A. W. Lipkowski (2002). "Direct antimicrobial properties of substance P." Life Sci **71**(7): 747-750.

Krause, A., R. Sillard, B. Kleemeier, E. Klüver, E. Maronde, J. R. Conejo-García, W. G. Forssmann, P. Schulz-Knappe, M. C. Nehls, F. Wattler, S. Wattler and K. Adermann (2003). "Isolation and biochemical characterization of LEAP-2, a novel blood peptide expressed in the liver." Protein Sci **12**(1): 143-152.

Lambert, R. W., K. Campton, W. Ding, H. Ozawa and R. D. Granstein (2002). "Langerhans cell expression of neuropeptide Y and peptide YY." Neuropeptides **36**(4): 246-251.

Lim, K. P. H., P. Milne, M. Poidinger, K. Duan, H. Lin, N. McGovern, H. Abhyankar, D. Zinn, T. M. Burke, O. S. Eckstein, R. Chakraborty, A. Sengal, B. Scull, E. Newell, M. Merad, K. L. McClain, T. K. Man, F. Ginhoux, M. Collin and C. E. Allen (2020). "Circulating CD1c+ myeloid dendritic cells are potential precursors to LCH lesion CD1a+CD207+ cells." Blood Adv **4**(1): 87-99.

Lu, Q., L. P. Samaranayake, R. P. Darveau and L. Jin (2005). "Expression of human beta-defensin-3 in gingival epithelia." J Periodontal Res **40**(6): 474-481.

Luger, T. A., T. Scholzen and S. Grabbe (1997). "The role of alpha-melanocyte-stimulating hormone in cutaneous biology." J Investig Dermatol Symp Proc **2**(1): 87-93.

Maerki, C., S. Meuter, M. Liebi, K. Mühlemann, M. J. Frederick, N. Yawalkar, B. Moser and M. Wolf (2009). "Potent and broad-spectrum antimicrobial activity of CXCL14 suggests an immediate role in skin infections." J Immunol **182**(1): 507-514.

McGovern, N., A. Schlitzer, M. Gunawan, L. Jardine, A. Shin, E. Poyner, K. Green, R. Dickinson, X. N. Wang, D. Low, K. Best, S. Covins, P. Milne, S. Pagan, K. Aljefri, M. Windebank, D. Miranda-Saavedra, A. Larbi, P. S. Wasan, K. Duan, M. Poidinger, V. Bigley, F. Ginhoux, M. Collin and M. Haniffa (2014). "Human dermal CD14⁺ cells are a transient population of monocyte-derived macrophages." Immunity **41**(3): 465-477.

Meller, S., J. Di Domizio, K. S. Voo, H. C. Friedrich, G. Chamilos, D. Ganguly, C. Conrad, J. Gregorio, D. Le Roy, T. Roger, J. E. Ladbury, B. Homey, S. Watowich, R. L. Modlin, D. P. Kontoyiannis, Y. J. Liu, S. T. Arold and M. Gilliet (2015). "T(H)17 cells promote microbial killing and innate immune sensing of DNA via interleukin 26." Nat Immunol **16**(9): 970-979.

Park, S. C., J. C. Moon, S. Y. Shin, H. Son, Y. J. Jung, N. H. Kim, Y. M. Kim, M. K. Jang and J. R. Lee (2016). "Functional characterization of alpha-synuclein protein with antimicrobial activity." Biochem Biophys Res Commun **478**(2): 924-928.

Pilkington, S. M., R. J. Dearman, I. Kimber and C. E. M. Griffiths (2018). "Langerhans cells express human β-defensin 3: relevance for immunity during skin ageing." Br J Dermatol **179**(5): 1170-1171.

Polak, M. E., S. M. Thirdborough, C. Y. Ung, T. Elliott, E. Healy, T. C. Freeman and M. R. Ardern-Jones (2014). "Distinct molecular signature of human skin Langerhans cells denotes critical differences in cutaneous dendritic cell immune regulation." J Invest Dermatol **134**(3): 695-703.

Raschig, J., D. Mailänder-Sánchez, A. Berscheid, J. Berger, A. A. Strömstedt, L. F. Courth, N. P. Malek, H. Brötz-Oesterhelt and J. Wehkamp (2017). "Ubiquitously expressed Human Beta Defensin 1 (hBD1) forms bacteria-entrapping nets in a redox dependent mode of action." PLoS Pathog **13**(3): e1006261.

Renn, C. N., D. J. Sanchez, M. T. Ochoa, A. J. Legaspi, C. K. Oh, P. T. Liu, S. R. Krutzik, P. A. Sieling, G. Cheng and R. L. Modlin (2006). "TLR activation of Langerhans cell-like dendritic cells triggers an antiviral immune response." J Immunol **177**(1): 298-305.

Rhodes, J. W., R. A. Botting, K. M. Bertram, H. Rana, H. Baharlou, E. E. Longmuir-Vine, P. Vegh, J. Fletcher, T. R. O’Neil, G. P. Parnell, J. D. Graham, N. Nasr, J. J. K. Lim, L. Barnouti, P. Haertsch, M. P. Gosselink, A. Di Re, G. Ctercteko, G. J. Jenkins, A. J. Brooks, E. Patrick, S. N. Byrne, M. A. Haniffa, A. L. Cunningham and A. N. Harman (2020). "Identification of HIV-Transmitting Sub-Epithelial Mononuclear Phagocytes in Human Anogenital and Colorectal Tissues." bioRxiv: 2020.2005.2026.117408.

Ross, R., X. L. Ross, H. Ghadially, T. Lahr, J. Schwing, J. Knop and A. B. Reske-Kunz (1999). "Mouse langerhans cells differentially express an activated T cell-attracting CC chemokine." J Invest Dermatol **113**(6): 991-998.

Sass, V., T. Schneider, M. Wilmes, C. Körner, A. Tossi, N. Novikova, O. Shamova and H. G. Sahl (2010). "Human beta-defensin 3 inhibits cell wall biosynthesis in Staphylococci." Infect Immun **78**(6): 2793-2800.

Schaerli, P., L. Ebert, K. Willimann, A. Blaser, R. S. Roos, P. Loetscher and B. Moser (2004). "A skin-selective homing mechanism for human immune surveillance T cells." J Exp Med **199**(9): 1265-1275.

Sinha, S., M. K. Harioudh, R. P. Dewangan, W. J. Ng, J. K. Ghosh and S. Bhattacharjya (2018). "Cell-Selective Pore Forming Antimicrobial Peptides of the Prodomain of Human Furin: A Conserved Aromatic/Cationic Sequence Mapping, Membrane Disruption, and Atomic-Resolution Structure and Dynamics." ACS Omega **3**(11): 14650-14664.

Sirvent, S., A. F. Vallejo, J. Davies, K. Clayton, Z. Wu, J. Woo, J. Riddell, V. K. Chaudhri, P. Stumpf, L. A. Nazlamova, G. Wheway, M. Rose-Zerilli, J. West, M. Pujato, X. Chen, C. H. Woelk, B. MacArthur, M. Ardern-Jones, P. S. Friedmann, M. T. Weirauch, H. Singh and M. E. Polak (2020). "Genomic programming of IRF4-expressing human Langerhans cells." Nat Commun **11**(1): 313.

Széles, L., S. Póliska, G. Nagy, I. Szatmari, A. Szanto, A. Pap, M. Lindstedt, S. J. Santegoets, R. Rühl, B. Dezsö and L. Nagy (2010). "Research resource: transcriptome profiling of genes regulated by RXR and its permissive and nonpermissive partners in differentiating monocyte-derived dendritic cells." Mol Endocrinol **24**(11): 2218-2231.

Yang, D., Q. Chen, D. M. Hoover, P. Staley, K. D. Tucker, J. Lubkowski and J. J. Oppenheim (2003). "Many chemokines including CCL20/MIP-3alpha display antimicrobial activity." J Leukoc Biol **74**(3): 448-455.

Zheng, H., H. Li, J. Zhang, H. Fan, L. Jia, W. Ma, S. Ma, S. Wang, H. You, Z. Yin and X. Li (2020). "Serum amyloid A exhibits pH dependent antibacterial action and contributes to host defense against Staphylococcus aureus cutaneous infection." J Biol Chem **295**(9): 2570-2581.
